# Swespine: the Swedish spine register: The 2012 report

**DOI:** 10.1007/s00586-013-2758-9

**Published:** 2013-04-11

**Authors:** Björn Strömqvist, Peter Fritzell, Olle Hägg, Bo Jönsson, Bengt Sandén, Swedish Society of Spinal Surgeons

**Affiliations:** 1 Department of Orthopedics Clinical Sciences Lund, Lund University Hospital, SE 22185 Lund, Sweden; 2 Department of Neuro-Orthopedics, Ryhov County Hospital Jönköping, Jönköping, Sweden; 3 Spine Center Göteborg, Gothenburg, Sweden; 4 Department of Orthopedics, University Hospital, Uppsala, Sweden

**Keywords:** Spine surgery, Outcome, Register, Disc herniation, Spondylolisthesis, Spinal stenosis

## Abstract

**Introduction:**

Swespine, the Swedish National Spine Register, has existed for 20 years and is in general use within the country since over 10 years regarding degenerative lumbar spine disorders. Today there are protocols for registering all disorders of the entire spinal column.

**Materials and methods:**

Patient-based pre- and postoperative questionnaires, completed before surgery and at 1, 2, 5 and 10 years postoperatively. Among patient-based data are VAS pain, ODI, SF-36 and EQ-5D. Postoperatively evaluation of leg and back pain as compared to preoperatively ("global assessment"), overall satisfaction with outcome and working conditions are registered in addition to the same parameters as preoperatively evaluation. A yearly report is produced including an analytic part of a certain topic, in this issue disc prosthesis surgery.

More than 75,000 surgically treated patients are registered to date with an increasing number yearly. The present report includes 7,285 patients; 1-, 2- and 5-year follow-up data of previously operated patients are also included for lumbar disorders as well as for disc prosthesis surgery.

**Results:**

For the degenerative lumbar spine disorders (disc herniation, spinal stenosis, spondylolisthesis and DDD) significant improvements are seen in all aspects as exemplified by pronounced improvement regarding EQ-5D and ODI. Results seem to be stable over time. Spinal stenosis is the most common indication for spine surgery. Disc prosthesis surgery yields results on a par with fusion surgery in disc degenerative pain. The utility of spine surgery is well documented by the results.

**Conclusion:**

Results of spine surgery as documented on a national basis can be utilized for quality assurance and quality improvement as well as for research purposes, documenting changes over time and bench marking when introducing new surgical techniques. A basis for international comparisons is also laid.

## Introduction

This report was written in autumn 2012, as we celebrated the 20th anniversary of the inception of the spine register. Historically, the register was introduced in 1992 at the state-of-the-art meeting, “The Degenerative Lumbar Spine” in Lund during an evaluation symposium led by Gunnar Andersson. At that time, the register involved a short form completed by doctors, and was presented in Acta Orthopaedica Scandinavica 1993 (Strömqvist and Jönsson 1993). Prospective data registration was not common then and was enthusiastically welcomed by the majority of spine surgeons in Sweden. However, only 4–6 departments actually began recording data in the early years during the mid-1990s. Consequently, Peter Fritzell, Olle Hägg, Bo Jönsson and Björn Strömqvist, who were all interested in establishing a register, formed a group to analyze the problems and suggest improvements. In the late 1990s, responsibility for the spine register was transferred to the Swedish Society of Spinal Surgeons (4 s), the current owner of what is now known as the Swedish Spine Register/Swespine. A largely patient-based online registration form was designed to address preoperative and postoperative variables. In addition, the coordinators/secretaries Carina Blom and Lena Oreby developed and provided on-line support services over time; and it is fair to say that without this organization and without their efforts, the register would not be what it is today.

These modifications, together with the conclusion that the register database should be stored on an “independent server”, that simplifications are crucial, and that physicians should be involved in the actual registering work as little as possible but instead be responsible for the analyses, reports and register-based improvement projects, changed the scene. In the late 1990s, the number of participating departments increased, and is currently varying between 35 and 39 of 42–45 departments providing spinal surgery services in Sweden (90 % coverage).

This Annual 2012 Register Report contains, in addition to a default presentation of updated FU-results from all spinal procedures covering degenerative disorders, an analysis specifically focused on total disc replacement (TDR).

Previous reports have specifically discussed for example,

- Spinal stenosis (http://www.4s.nu/pdf/Report_2007_englishversion.pdf)
- Disc herniation (http://www.4s.nu/pdf/Ryggregisterrapport_2008_eng_version.pdf, and http://www.4s.nu/pdf/Report_2010_Swespine_Englishversion.pdf)
- Isthmic spondylolisthesis (http://www.4s.nu/pdf/Report_2011_Swespine_Englishversion.pdf)
- Segmental pain/DDD (http://www.4s.nu/pdf/Englishversion%20_report2009.pdf).

Our goal is to present baseline and FU data from all diagnostic groups. Today, only degenerative lumbar spine procedures are presented in large quantities, but for all other diagnostic entities and associated procedures, we need larger quantities of data to make similar evaluations as for degenerative lumbar spine surgery. However, the number of cervical spine procedures is growing, with interesting results.

Once again, the mega effort by registering surgeons, secretaries and patients has resulted in a comprehensive annual report from Swespine.

The disc replacement analysis in this report answers some questions, while raising others and we will return to this subject in the future. As the quantity of data from other diagnostic entities grows, their contribution will make the Swespine register even more interesting.

The number of procedures entered in the register has set a new record in 2011, i.e. 7,500 lumbar spine procedures out of approximately 10,000 procedures performed annually in the country, while the follow-up rate remains largely unchanged or 75–80 % on a national scale. Through a recently launched National Register Center, which will assist with collection and entry of follow-up data, it is our top priority to further improve the credibility of data presentation as well as the rate of follow-up.

## Preoperative and surgical data on lumbar spine procedures

The preoperative data entered into the Swespine protocol are entirely patient-based, including age, sex, smoking habits, duration of back and leg pain before surgery, consumption of analgesics, walking distance, back and leg pain on the VAS scale, health-related quality of life as documented by the SF-36 and EQ-5D and spine-related disability as documented by the Oswestry disability Index, ODI. This means that the protocol mainly relies on PROM data (patient reported outcome measurements).

The surgical data are the only data completed by the surgeon at the time of discharge from hospital, and include diagnosis, procedure, implant (if any), hospitalization time, antibiotic prophylaxis and occurrence of complications.

At follow-up, the same data (PROM) as registered at baseline are completed and also patient-based evaluation of leg and back pain as compared to preoperatively (“global assessment”) of outcome, meaning that the patient reports the change with respect to the indication for surgery (for example change in leg pain in LDH-patients). Overall satisfaction with outcome (satisfied, undecided, dissatisfied) also was graded by the patients.

The group “spondylolisthesis” refers to patients with isthmic spondylolisthesis.

In this report, a total of 7,208 patients who had had lumbar spine surgery for different diagnoses, at a total of 43 departments, were entered in the register in 2011. The corresponding figure for 2010 was 6,992 patients from 38 departments.

**Fig. 1:**
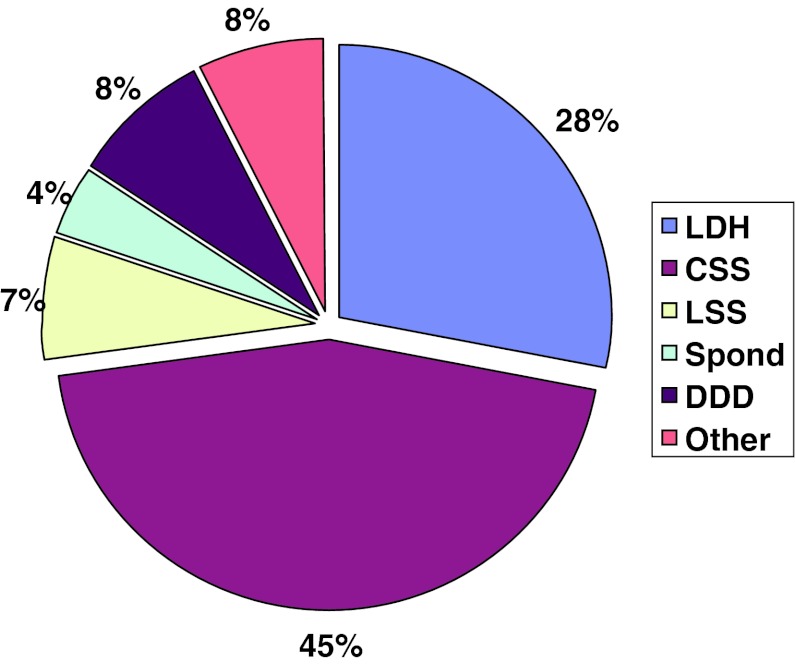
Breakdown by diagnosis in the total material 2011, 7,529 patients

The distribution of diagnoses for patients operated in 2011 was as follows: disc herniation 28 %, central spinal stenosis 45 %, lateral spinal stenosis 7 %, spondylolisthesis 4 %, segmental pain/DDD (disc degenerative disorder) 8 % and other 8 %, see Fig. 1.

Diagnosis-related patient demographics and surgical data are presented below.

### Disc herniation

#### Demographic data

**Fig. 2:**
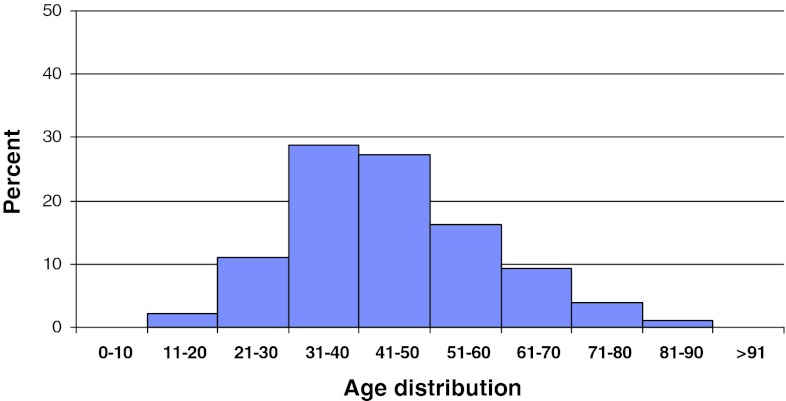
Distribution by age, disc herniation, *n* = 2,118

In 2011, 2,118 patients operated for lumbar disc herniation were registered in Swespine. There were 55 % men and 45 % women. The proportion of smokers was 17 %. The mean age was 45 (15–91) years, Fig. 2. However, the median age was 40, meaning that more elderly patients were operated than younger.

For 88 % of patients, this discectomy was their first lumbar spine surgery, while 12 % had been previously operated.

**Fig. 3:**
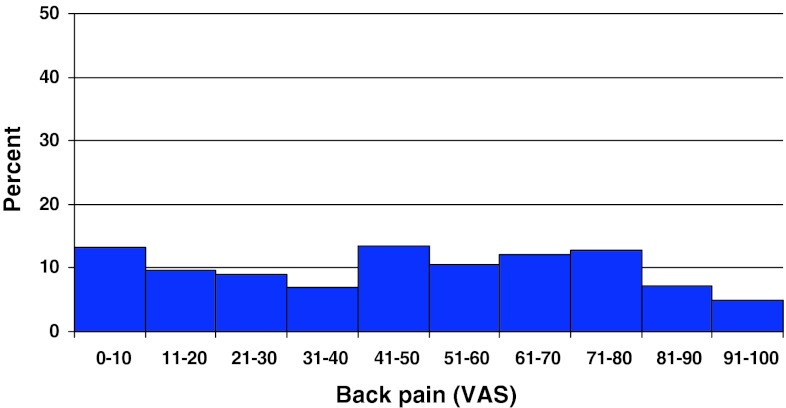
Back pain on the VAS preoperatively in patients with disc herniation (%)

**Fig. 4:**
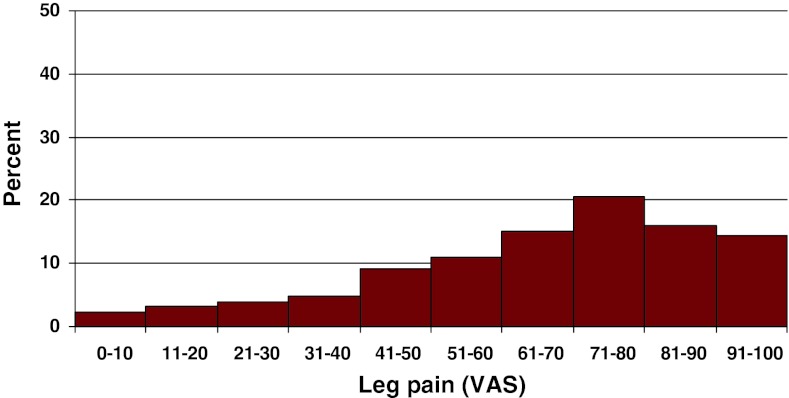
Leg pain on the VAS preoperatively in patients with disc herniation (%)

Preoperative duration of back pain was as follows: 6 % reported no back pain, 11 % had a history of less than 3 months of back pain, 48 % 3–12 months, 15 % 1–2 years and 20 % more than 2 years. Preoperative duration of leg pain/sciatica was as follows: 1 % reported no leg pain, 16 % had leg pain for less than 3 months, 55 % for 3–12 months, 16 % for 1–2 years and 16 % had pain for more than 2 years. Mean back pain on the visual analog scale (VAS) was 48 with a spread from 0 to 100, while mean leg pain/sciatica on the VAS was 67 with the same spread from 0 to 100. Distribution regarding both back and leg pain can be seen in Figs. 3 and 4.

Regular analgesic use was reported by 64 % of patients, intermittent use by 26 %, while 10 % reported that they did not take any form of analgesics.

Walking distance was estimated at less than 100 m by 31 % of patients, 100–500 m by 23 % of patients, 500 m–1 km for 15 % of patients and more than 1 km by 31 % of patients.

#### Surgical data

Conventional discectomy was carried out in 45 % of cases and microscopic discectomy in 41 %. The remaining procedures consisted of various combinations mainly involving decompressive surgery for patients with disc herniation with spinal stenosis. Mean length of stay in days, i.e., time from surgery through discharge, was 2.73 (0–22).

### Central spinal stenosis

#### Demographic data

**Fig. 5:**
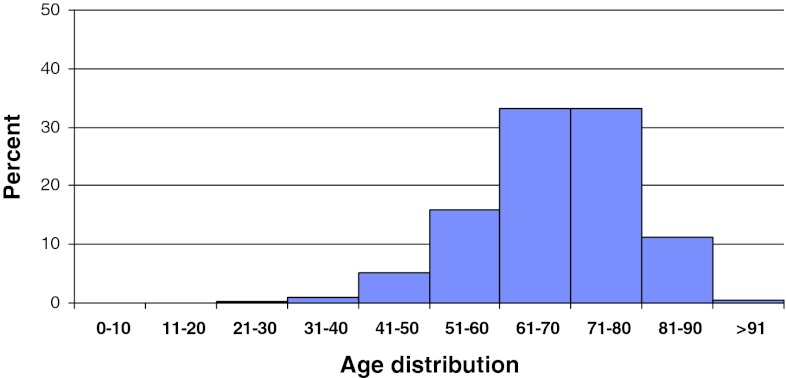
Distribution by age, central spinal stenosis, *n* = 3,367 patients

A total of 3,367 patients were registered for operations for central spinal stenosis in 2011. The patients included 44 % men and 56 % women. Mean age was 68 (23–95) years. Figure 5 shows the age distribution.

The proportion of smokers was 10 %. For 79 % of patients, this operation was their first surgery, while 21 % had been previously operated one to three times.

Preoperative duration of back pain was as follows: 5 % reported no back pain, 2 % had a history of less than 3 months of back pain, 16 % 3–12 months, 23 % 1–2 years and 55 % more than 2 years. Regarding leg pain, 4 % of patients reported no leg pain, 2 % of patients with central spinal stenosis reported leg problems for less than 3 months, 24 % for 3–12 months, 29 % for 1–2 years and 41 % reported problems for more than 2 years.

**Fig. 6:**
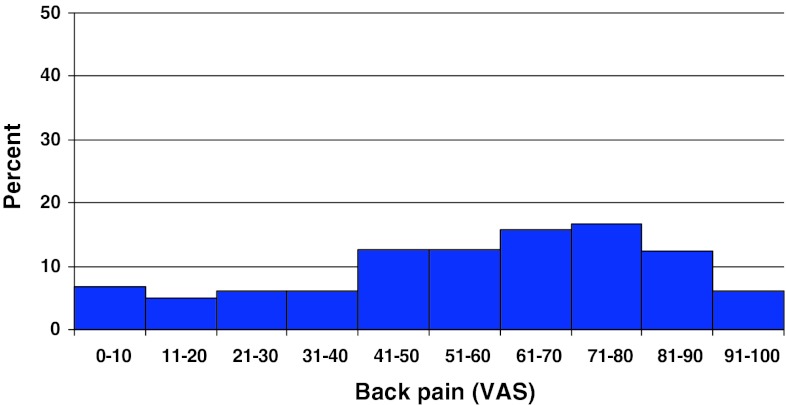
Back pain on the VAS preoperatively in patients with central spinal stenosis (%)

**Fig. 7:**
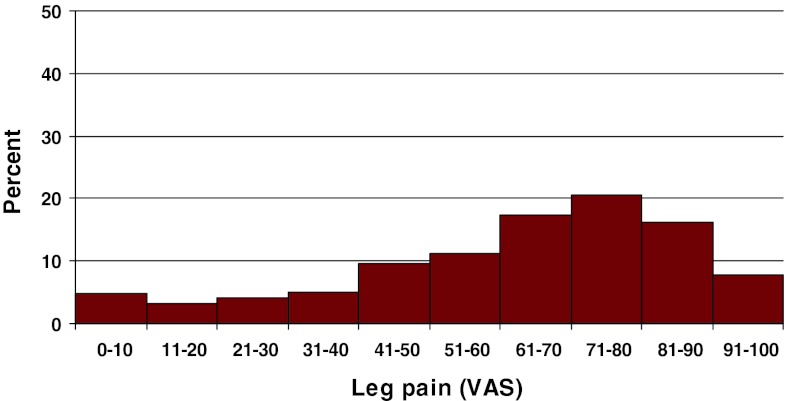
Leg pain on the VAS preoperatively in patients with central spinal stenosis (%)

Mean back pain on the VAS in the group was 58 (0–100) and mean leg pain/sciatica (VAS) 63 (0–100). Figures 6 and 7 present the distribution of reported VAS.

Of patients with central spinal stenosis, 55 % reported regular use of analgesics, 29 % reported intermittent use and 15 % reported that they did not take any analgesic medication.

Walking distance was estimated at less than 100 m by 40 % of patients, 100–500 m by 31 % of patients, 500 m–1 km for 15 % of patients and more than 1 km by 14 % of patients.

#### Surgical data

72 % of the patients had decompressive surgery as the sole procedure, in 52 % conventional surgery and in 21 % of cases microscopic surgery. Decompression combined with posterior instrumented fusion was carried out in 20 % of the patients, decompression + posterior non-instrumented fusion in 3 %, decompression + TLIF in 1 % and other procedures in 4 %. Mean length of stay in days was 4.31 (0–29).

### Lateral spinal stenosis

#### Demographic data

**Fig. 8:**
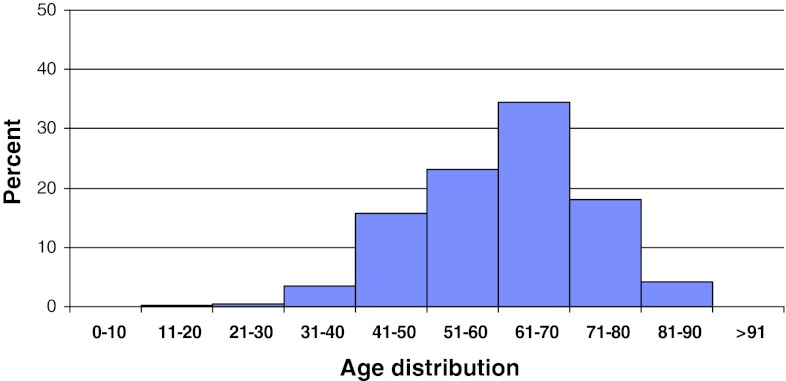
Distribution by age, lateral spinal stenosis, *n* = 532

During the year, 532 patients were operated for lateral spinal stenosis. The patients included 52 % men and 49 % women. The group included 16 % smokers. Mean age was 61 (18–88) years; Fig. 8 shows the age distribution.

The majority of patients with lateral spinal stenosis, 75 %, had had no previous spine surgery while 25 % had been operated on one or more times before the current procedure.

**Fig. 9:**
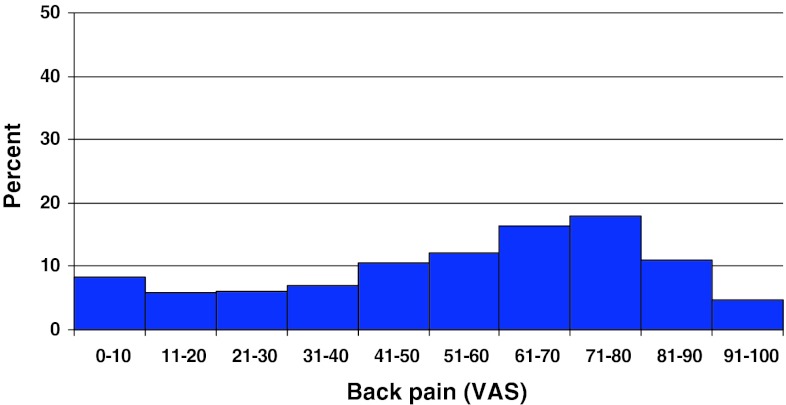
Back pain on the VAS preoperatively in patients with lateral spinal stenosis (%)

**Fig. 10:**
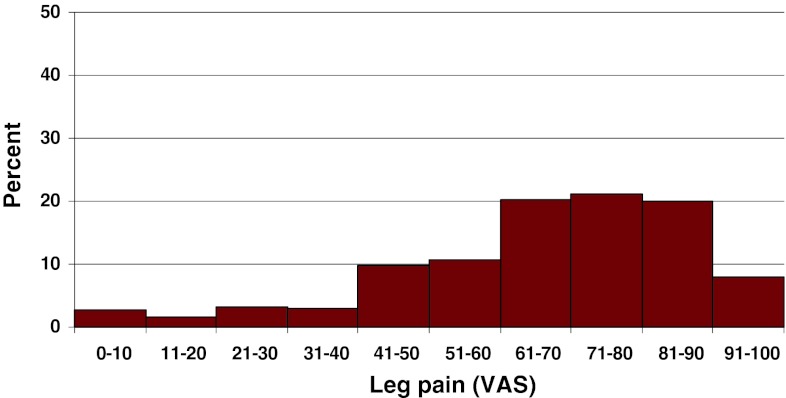
Leg pain on the VAS preoperatively in patients with lateral spinal stenosis (%)

Preoperative duration of back pain was as follows: 6 % reported no back pain, 2 % had a history of less than 3 months of back pain, 19 % 3–12 months, 18 % 1–2 years and 54 % more than 2 years. Regarding leg pain, 1 % of patients with lateral spinal stenosis reported no leg pain, 2 % of patients reported leg problems for less than 3 months, 27 % for 3–12 months, 29 % for 1–2 years and 41 % reported problems for more than 2 years. Mean back pain on the VAS in the group was 56 (0–100) and mean leg pain (VAS) 67 (0–100). Figures 9 and 10 present the distribution of reported VAS.

Regular analgesic use was reported by 60 % of patients, intermittent use by 29 %, and 12 % reported that they did not take any analgesics. The majority of patients reported limited walking ability, 28 % reported that they were able to walk less than 100 m, 32 % were able to walk 100–500 m, 20 % 500 m–1 km and 20 % had a walking distance of more than 1 km.

#### Surgical data

Decompression surgery was the type of procedure in the majority of cases, 72 %, including 49 % conventional, 23 % microscopic decompression, 18 % had decompression + posterior instrumented fusion and 3 % decompression + TLIF. Mean length of stay (total) was 3.5 (0–23) days.

### Spondylolisthesis

#### Demographic data

**Fig. 11:**
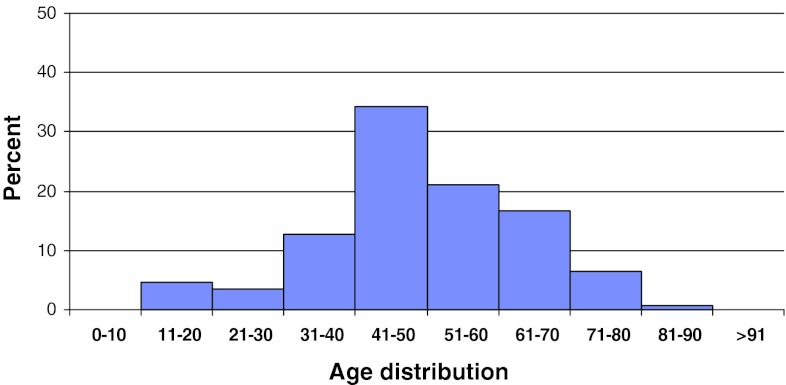
Distribution by age, spondylolisthesis, *n* = 323 patients

A total of 323 patients, including 47 % men and 53 % women, were reported for 2011. This group included 12 % smokers. Mean age was 50 (14–82) years and Fig. 11 shows the age distribution.

For 89 % of patients, the current procedure was the first time they had surgery on the lumbar spine, while the remainder had one or two previous procedures.

Preoperative duration of back pain was as follows: 2 % reported no back pain, 1 % had a history of less than 3 months of back pain, 11 % 3–12 months, 19 % 1–2 years and 66 % more than 2 years. Regarding leg pain, 6 % of patients with spondylolisthesis reported no leg pain, 1 % reported leg problems for less than 3 months, 18 % 3–12 months, 29 % 1–2 years and 47 % reported problems for more than 2 years.

**Fig. 12:**
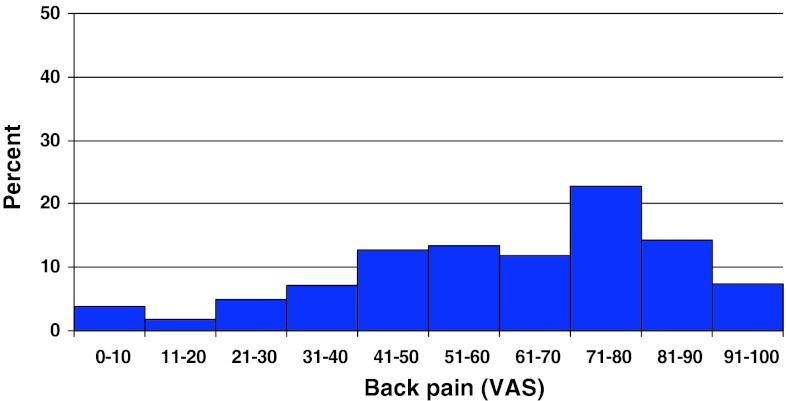
Back pain on the VAS preoperatively in patients with spondylolisthesis (%)

**Fig. 13:**
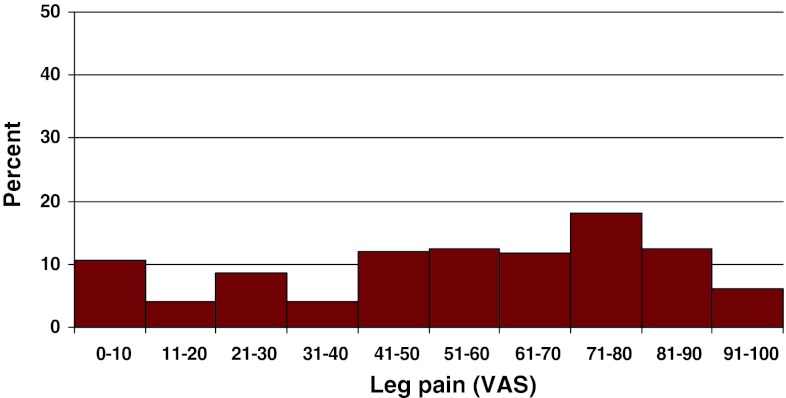
Leg pain on the VAS in patients with spondylolisthesis (%)

Preoperative lumbar pain on the VAS was 62 (0–100) and preoperative leg pain was 55 (0–99). Figures 12 and 13 present the distribution of pain on the VAS.

Regular analgesic use was reported by 48 % of patients, intermittent use by 37 %, while 14 % did not use analgesics.

Walking distance was estimated to less than 100 m by 22 % of patients, 100–500 m by 24 % of patients, 500 m–1 km by 20 % of patients and more than 1 km by 34 % of patients.

#### Surgical data

Patients with spondylolisthesis had a variety of different procedures. They are presented in descending order of frequency: decompression + instrumented fusion 53 %, posterior instrumented fusion 15 %, PLIF with or without foreign implant 14 %, decompression + TLIF 4 %, decompression + non-instrumented fusion 3 %, decompression + PLIF 1 %, posterior non-instrumented fusion 1 % and decompressive interventions in the remaining cases. Mean length of stay in days was 5.54 (1–27).

### Degenerative disc disorder (DDD)/segmental pain

#### Demographic data

**Fig. 14:**
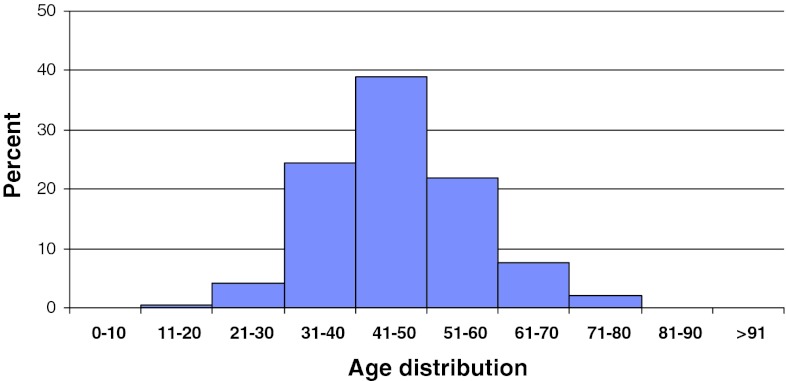
Distribution by age, DDD, *n* = 620 patients

A total of 620 patients were registered for surgical intervention for DDD in 2011, including 43 % men and 57 % women. The proportion of smokers was 11 %. Mean age was 47 (16–80) years; Fig. 14 shows the age distribution.

In this group of patients, 68 % had lumbar spine surgery for the first time, while 32 % had been operated one or more times previously.

Preoperative duration of back pain in patients with DDD was as follows: 0.4 % reported no back pain, 0.2 % had a history of less than 3 months of back pain, 9 % 3–12 months, 16 % 1–2 years and 75 % more than 2 years. Regarding leg pain, 18 % of patients with DDD reported no leg pain, 2 % reported leg problems for less than 3 months, 16 % 3–12 months, 18 % 1–2 years and 47 % reported problems for more than 2 years.

**Fig. 15:**
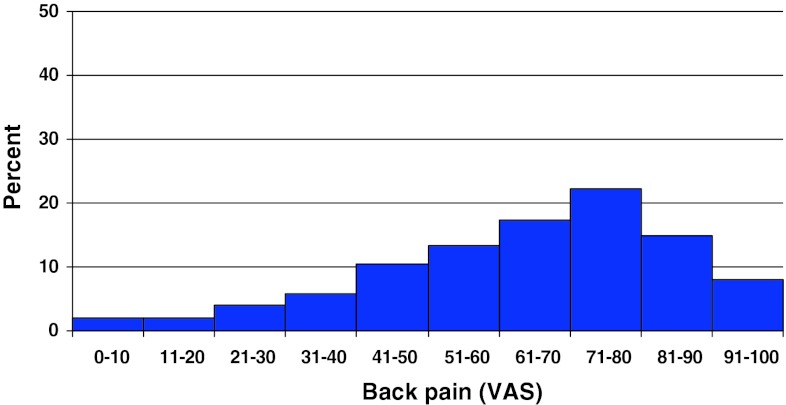
Back pain on the VAS preoperatively in patients with DDD (%)

**Fig. 16:**
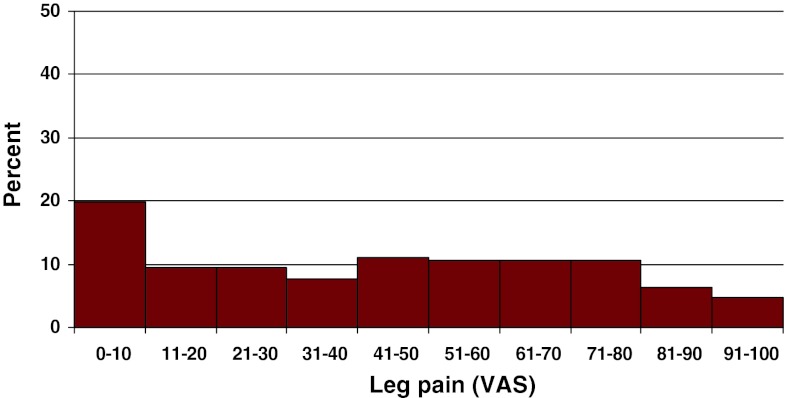
Leg pain on the VAS preoperatively in patients with DDD (%)

Estimation on the VAS scale for back pain showed a mean of 65 (0–100) and leg pain, 43 (0–100). Figures 15 and 16 present the distribution of pain on the VAS.

Regular analgesic use was reported by 61 % of patients, intermittent use by 31 %, while 8 % never took analgesics.

Walking distance was estimated at less than 100 m by 15 % of patients, 100–500 m by 21 % of patients, 500 m–1 km by 19 % of patients and more than 1 km by 45 % of patients.

#### Surgical data

A heterogenous surgical treatment spectrum was also seen for this diagnosis as follows: posterior instrumented fusion 29 %, PLIF 18 %, disc replacement 18 %, decompression + posterior instrumented fusion 14 %, TLIF 5 %, decompression + TLIF 5 %, decompression + PLIF 4 %, ALIF with instrument 2 %, posterior non-instrumented fusion 1 %, decompression + posterior non-instrumented fusion 1 % and a smaller quantity in other interventions. Mean length of stay was 5.08 (1–18) days.

## One-year follow-up of lumbar spine procedures

A total of 7,051 patients were operated in 2010 and 5,124 (73 %) completed 1-year follow-up. The distribution is as follows: disc herniation 1,365, central spinal stenosis 2,412, lateral spinal stenosis 399, spondylolisthesis 259 and DDD 530. Patients with “other operations” (*n* = 159) are not presented in the following results.

### Disc herniation

Of 1,365 patients who were operated for lumbar disc herniation and completed 1-year follow-up, 56 % were men and 44 % women, with a mean age of 44 (13–90) years.

**Fig. 17:**
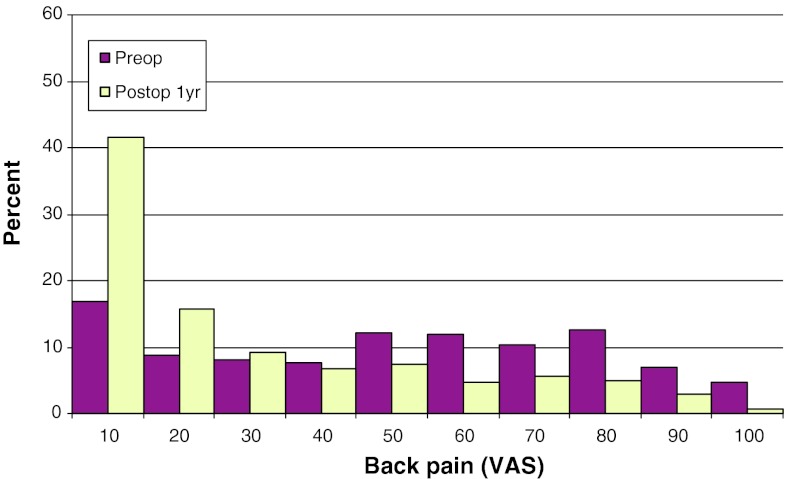
Back pain on the VAS preoperatively and 1 year postoperatively in patients operated for lumbar disc herniation in 2010 (%)

**Fig. 18:**
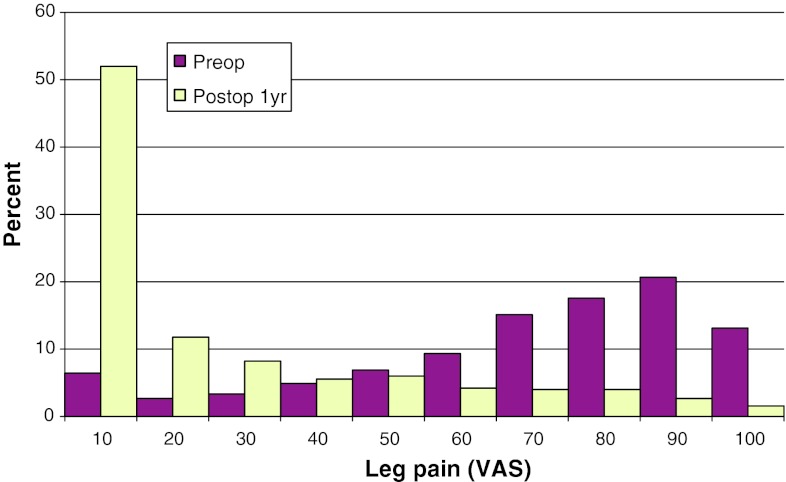
Leg pain on the VAS preoperatively and 1 year postoperatively in patients operated for lumbar disc herniation in 2010 (%)

Mean preoperative VAS for back pain was 46, compared with 26 at follow-up. The corresponding figures for leg pain were 67 preoperatively, and 22 at follow-up. Figures 17 and 18 show preoperative and postoperative VAS for back and leg pain, respectively.

Perceived improvement relating to back pain: completely pain-free 20 %, significantly improved 45 %, somewhat improved 17 %, unchanged 6 %, deteriorated 5 % and 7 % did not report preoperative back pain.

Perceived improvement relating to leg pain (global assessment): completely pain-free 35 %, significantly improved 37 %, somewhat improved 15 %, unchanged 6 %, deteriorated 5 % and 2 % did not report preoperative leg pain.

Overall patient satisfaction with surgical outcome: 78 % were satisfied, 15 % uncertain and 7 % dissatisfied.

Use of analgesics 1 year postoperatively: regular 17 %, intermittent 31 % and none 52 %.

Ability to walk 1 year postoperatively: <100 m 5 %, 100–500 m 8 %, 500 m–1 km 11 %, >1 km 76 %, a substantial improvement compared with preoperatively.

**Fig. 19:**
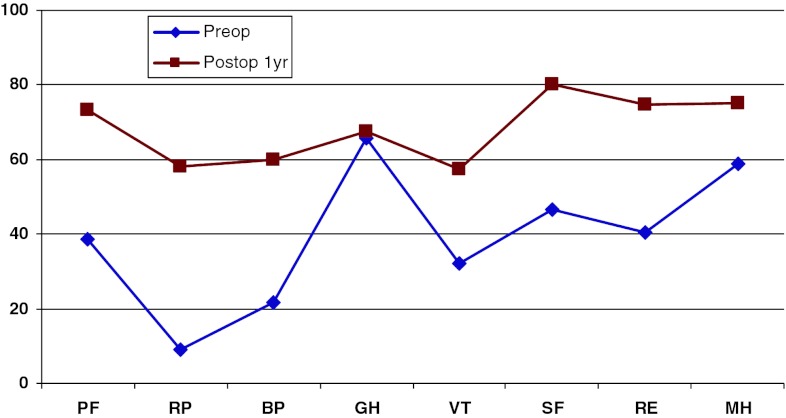
SF-36 preoperatively and 1 year postoperatively for patients operated for lumbar disc herniation in 2010

Figure 19 shows preoperative and 1-year postoperative status regarding health-related quality of life as measured with the SF-36. The improvement is significant in all domains except “General health”.

The results from the EQ-5D analysis are presented both as an EQ-5D index value, i.e. the answers of the five questions included in the questionnaire presented as an index value where 1 represents perfect quality of life and 0 represents “equal to death”, and also on the VAS scale, EQ-VAS, ranging from 0 to 100 where a high value is better. The results for lumbar disc herniation are as follows: the mean EQ-5D index value preoperatively was 0.26, and 1 year postoperatively it was 0.71. The mean EQ-VAS preoperatively was 46, and 1 year postoperatively it was 72.

### Central spinal stenosis

This group includes 2,412 patients, 45 % men and 55 % women, with a mean age of 68 (18–95) years.

**Fig. 20:**
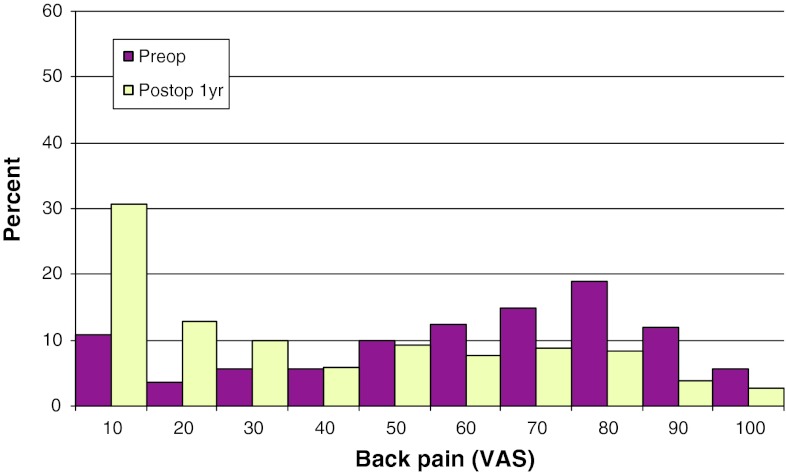
Back pain on the VAS preoperatively and 1 year postoperatively in patients operated for lumbar central spinal stenosis in 2010 (%)

**Fig. 21:**
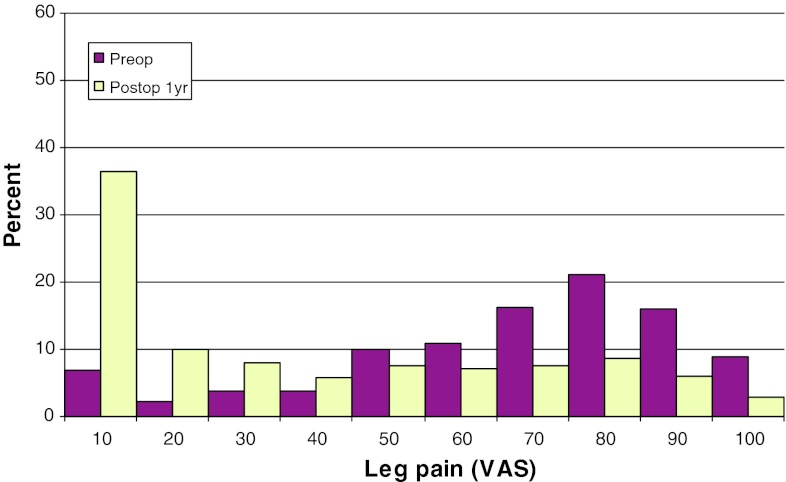
Leg pain on the VAS preoperatively and 1 year postoperatively in patients operated for lumbar central spinal stenosis in 2010 (%)

Mean preoperative VAS for back pain was 56, compared with 35 1 year postoperatively. The corresponding figures for leg pain were 63 and 34, respectively. Figures 20 and 21 show pre- and postoperative VAS for back and leg pain, respectively.

One year postoperatively, 16 % of patients felt they were completely pain-free, 36 % significantly improved, 18 % somewhat improved, 13 % unchanged, 9 % deteriorated with regard to back pain and 8 % reported no preoperative back pain. The corresponding figures for leg pain were 24 % completely pain-free, 29 % significantly improved, 18 % somewhat improved, 12 % unchanged and 11 % deteriorated and 7 % reported no preoperative leg pain.

Overall patient satisfaction with outcome of the procedure was as follows: 64 % were satisfied, 22 % uncertain and 13 % dissatisfied with the surgical outcome.

Analgesic use 1 year postoperatively: regular 31 %, intermittent 33 % and none 36 %.

Ability to walk 1 year postoperatively: <100 m 20 %, 100–500 m 21 %, 500 m–1 km 17 % and >1 km 42 %, which was a substantial improvement compared with preoperatively.

**Fig. 22:**
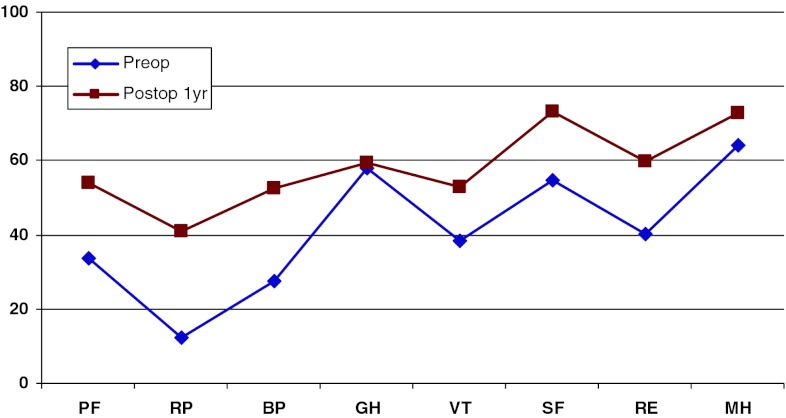
SF-36 preoperatively and 1 year postoperatively for patients operated for lumbar central spinal stenosis 2010

In addition, 1 year postoperatively, patients in the central spinal stenosis category demonstrated improvement of SF-36 score in all dimensions except “General health”. The improvement was less pronounced than in the disc herniation group, but was probably similar when adjusted for age, see Fig. 22.

The mean EQ-5D index value preoperatively: 0.35, and 1 year postoperatively 0.63. Mean EQ-VAS preoperatively (max 100): 48, 1 year postoperatively 64.

### Lateral spinal stenosis

This patient group included 335 patients, 50 % men and 50 % women, with a mean age of 61 (26–88) years.

**Fig. 23:**
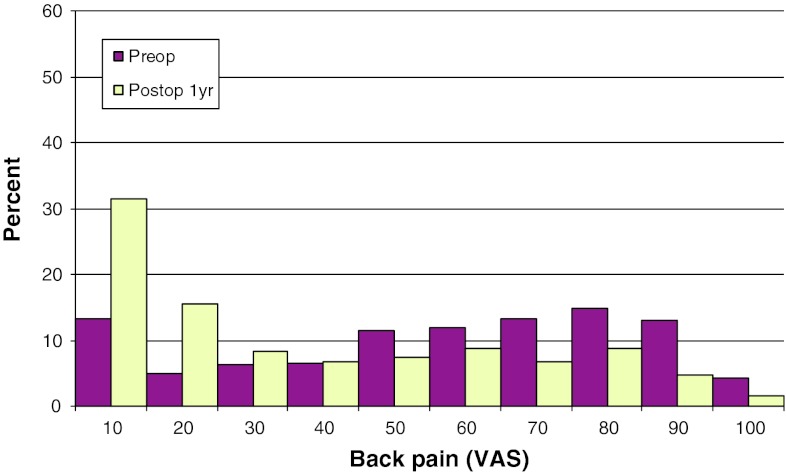
Back pain on the VAS preoperatively and 1 year postoperatively in patients operated for lumbar lateral spinal stenosis in 2010 (%)

**Fig. 24:**
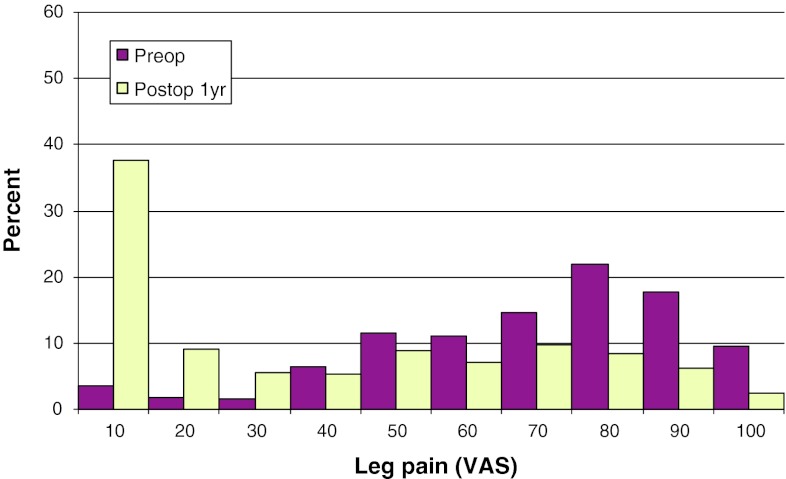
Leg pain on the VAS preoperatively and 1 year postoperatively in patients operated for lumbar lateral spinal stenosis in 2010 (%)

Mean preoperative VAS for back pain was 53, compared with 33 1 year postoperatively. The corresponding figures for leg pain were 65 and 34, respectively. Figures 23 and 24 show the distribution of pre- and postoperative VAS for back and leg pain.

One year postoperatively, 14 % of patients were completely pain-free, 33 % significantly improved, 22 % somewhat improved, 13 % unchanged, 11 % deteriorated with regard to back pain and 8 % reported no preoperative back pain. The corresponding figures for leg pain were 24 % completely pain-free, 30 % significantly improved, 21 % somewhat improved, 13 % unchanged, 9 % deteriorated and 3 % reported no preoperative leg pain.

Patient satisfaction with surgical outcome: 62 % satisfied, 25 % uncertain and 14 % dissatisfied.

Medication use 1 year postoperatively: 30 % regularly, 33 % intermittently and 38 % took no medication.

Ability to walk 1 year postoperatively: <100 m 15 %, 100–500 m 19 %, 500 m–1 km 17 % and >1 km 49 %.

**Fig. 25:**
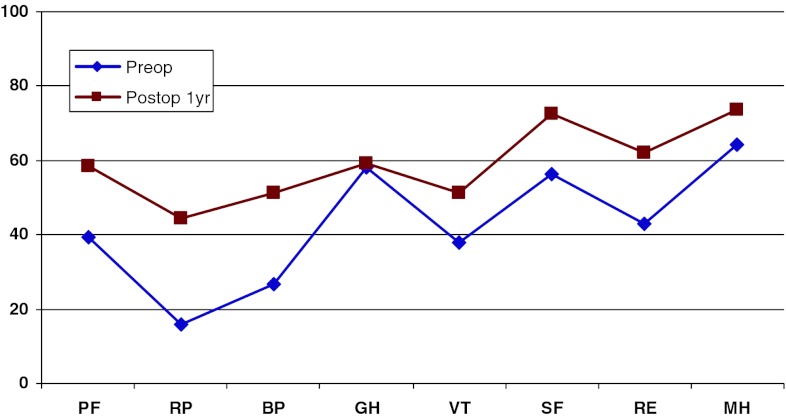
SF-36 preoperatively and 1 year postoperatively for patients operated for lumbar lateral spinal stenosis in 2010

The patient group operated for lateral spinal stenosis also showed improvement in SF-36 scores, though somewhat less pronounced, see Fig. 25.

The mean EQ-5D index value preoperatively was 0.35, and 1 year postoperatively 0.62. The mean EQ-VAS preoperatively was 47, and 1 year postoperatively 65.

### Spondylolisthesis

In all, 247 patients, 45 % men and 55 % women, operated during the period for spondylolisthesis completed 1-year follow-up. Mean age was 50 (11–83) years.

**Fig. 26:**
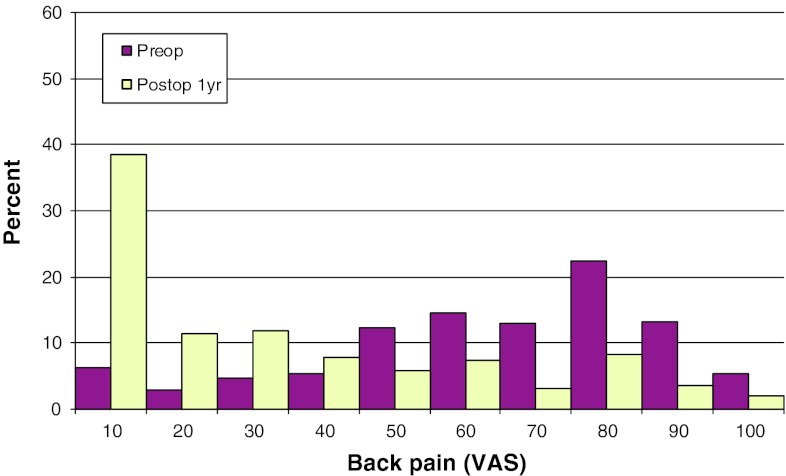
Back pain on the VAS preoperatively and 1 year postoperatively in patients operated for spondylolisthesis in 2010 (%)

**Fig. 27:**
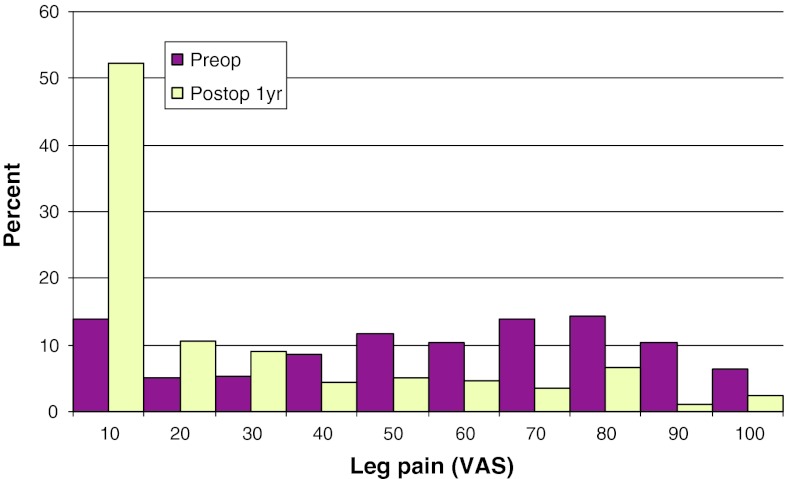
Leg pain on the VAS preoperatively and 1 year postoperatively in patients operated for spondylolisthesis in 2010 (%)

Mean preoperative VAS for back pain was 60, compared with 29 1 year postoperatively. The corresponding figures for leg pain were 52 and 23, respectively. Figures 26 and 27 show pre- and postoperative VAS for back and leg pain.

At the 1-year follow-up, 15 % of patients felt they were completely pain-free, 47 % significantly improved, 18 % somewhat improved, 9 % unchanged, 7 % deteriorated with regard to back pain and 4 % did not report back pain preoperatively. The corresponding figures for leg pain were 27 % completely pain-free, 39 % significantly improved, 13 % somewhat improved, 7 % unchanged, 6 % deteriorated and 9 % reported no preoperative leg pain.

Overall patient satisfaction with outcome of the operation: 73 % satisfied, 16 % uncertain and 11 % dissatisfied.

Regular intake of analgesics 1 year postoperatively was reported by 23 %, intermittent use by 32 % and no intake of analgesics at all by 45 %.

Ability to walk 1 year postoperatively: <100 m 7 %, 100–500 m 11 %, 500 m–1 km 13 % and >1 km 70 %, a substantial improvement compared with preoperatively.

**Fig. 28:**
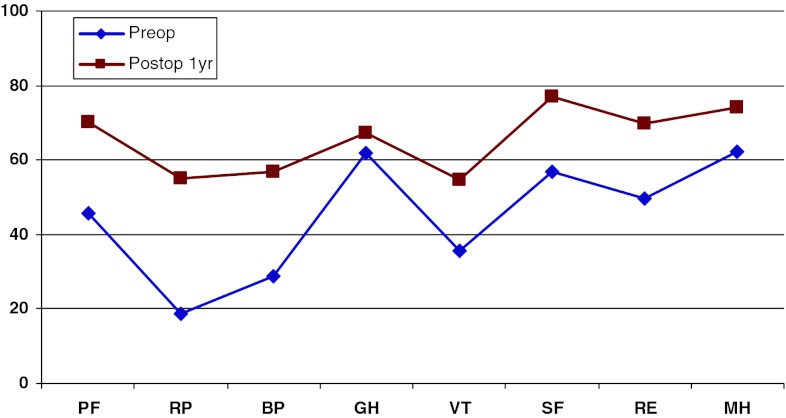
SF-36 preoperatively and 1 year postoperatively for patients operated for spondylolisthesis in 2010

Spondylolisthesis patients showed good improvement in their SF-36 scores 1 year postoperatively compared with preoperatively, see Fig. 28.

The mean value for EQ-5D preoperatively was 0.37, and 1 year postoperatively 0.69. The mean EQ-VAS preoperatively was 48, and 1 year postoperatively 68.

### DDD/segmental pain

In all, 1-year follow-up was completed by 518 patients, 48 % men and 52 % women, operated during the period. Mean age was 45 (18–80) years.

**Fig. 29:**
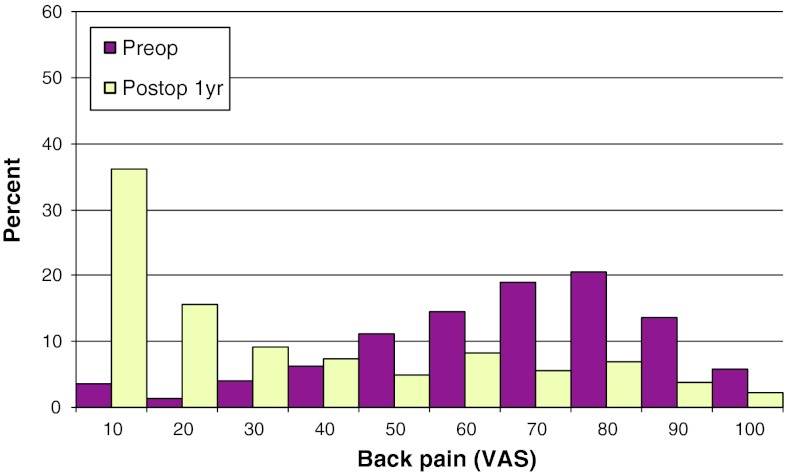
Back pain on the VAS preoperatively and 1 year postoperatively in patients operated for DDD in 2010 (%)

**Fig. 30:**
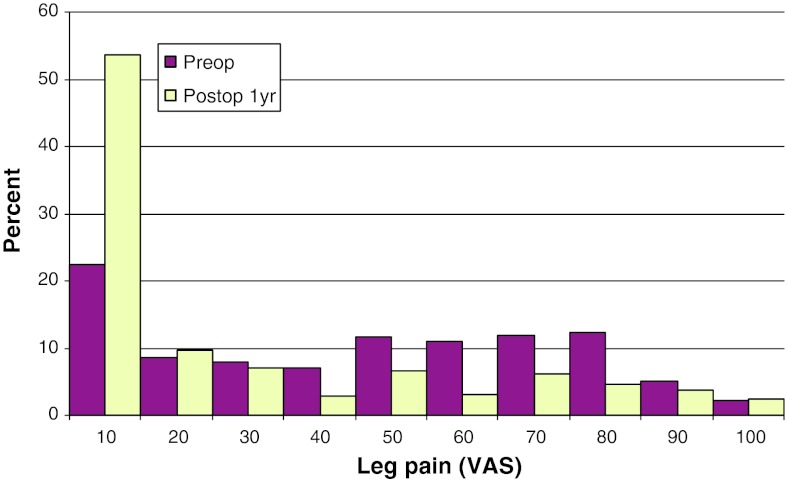
Leg pain on the VAS preoperatively and 1 year postoperatively in patients operated for DDD in 2010 (%)

Mean preoperative VAS for back pain was 62, compared with 30 1 year postoperatively. The corresponding figures for leg pain were 42 and 23, respectively. Figures 29 and 30 show pre- and postoperative VAS for back and leg pain.

One year postoperatively, patients operated for DDD perceived back pain as follows: completely pain-free 20 %, significantly improved 47 %, somewhat improved 17 %, unchanged 7 %, deteriorated 8 % and 1 % reported no back pain before surgery. The corresponding figures for leg pain: completely pain-free 26 %, significantly improved 28 %, somewhat improved 15 %, unchanged 7 %, deteriorated 9 % and 14 % reported no preoperative leg pain.

Regarding patient satisfaction with outcome of the operation: 74 % were satisfied, 14 % uncertain and 12 % dissatisfied.

Among these patients, 26 % took analgesics regularly 1 year postoperatively, 30 % did so intermittently and 44 % reported that they did not use any analgesics.

Ability to walk 1 year postoperatively: <100 m 6 %, 100–500 m 9 %, 500 m–1 km 13 % and >1 km 73 %, a substantial improvement compared with preoperatively.

**Fig. 31:**
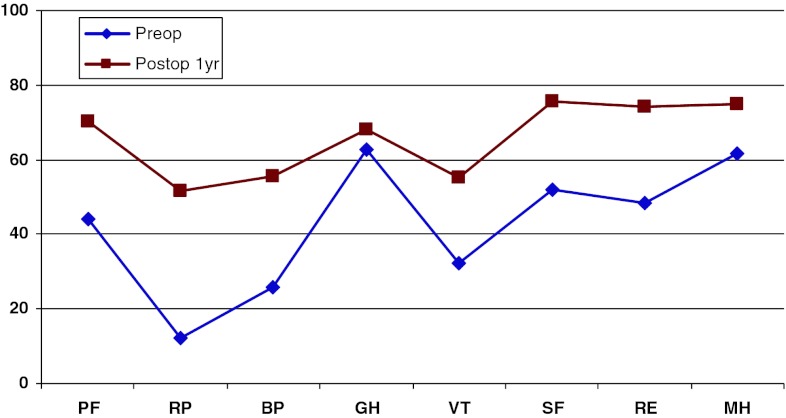
SF-36 preoperatively and 1 year postoperatively for patients operated for DDD in 2010

Figure 31 shows the pre- and postoperative SF-36 profiles for patients operated for DDD; the profiles are similar to the other diagnoses. Both the physical and mental domains show improvement.

The mean EQ-5D index value preoperatively was 0.33, and 1 year postoperatively 0.65. The mean EQ-VAS preoperatively was 44, and 1 year postoperatively 68.

### Oswestry disability index, ODI, before and 1 year after surgery for all diagnoses

**Fig. 32:**
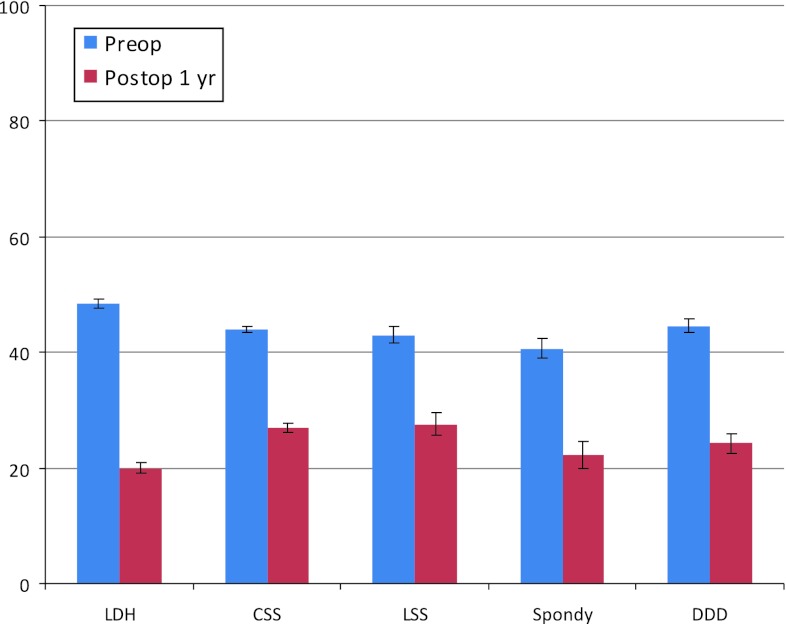
ODI score inclusive of before and one year after lumbar spine surgery, related to diagnosis, for patients operated in 2010 (mean ± CI)

Below is a comparison of pre- and postoperative “disability” as measured by the Oswestry index. All diagnoses show a significant reduction in measured functional limitation; most pronounced is disc herniation, see Fig. 32. A score of 0–20 is considered as no or little “disability”.

## Two-year follow-up of lumbar spine procedures

A total of 3,912 patients operated on in 2009 have completed preoperative, 1- and 2-year follow-up postoperative protocols. The most common diagnoses are disc herniation, 1,035 and central spinal stenosis, 1,907 patients. In all, 249 patients had been operated for lateral spinal stenosis, 1,202 for spondylolisthesis and 391 for DDD. The remaining 102 had other diagnoses. Below is a comparison of several parameters assessed at 1- and 2-year follow-up. Only patients who responded on all three occasions are included.

**Fig. 33:**
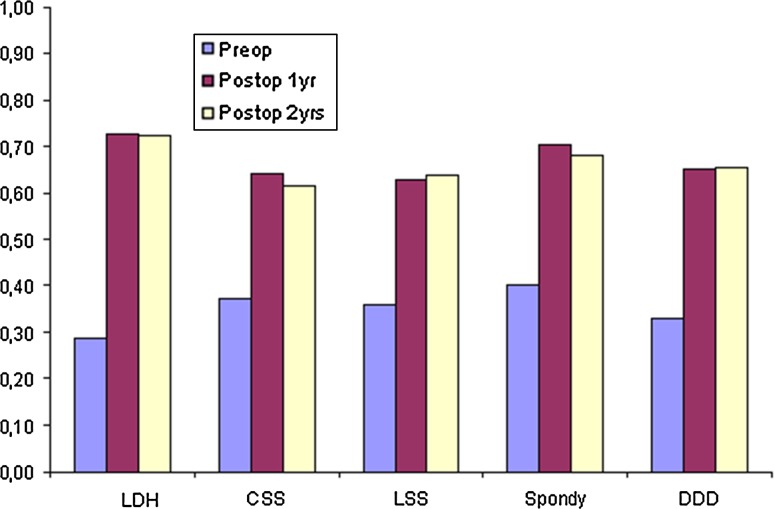
Quality of life preoperatively, 1 and 2 years postoperatively, as measured by EQ-5D. *LDH* lumbar disc herniation, *CSS* central spinal stenosis, *LSS* lateral spinal stenosis, *Spondy* spondylolisthesis, *DDD* degenerative disc disease

Table 1 presents pain on the VAS, diagnosis-related, over time. Tables 2, 3, 4, 5 and 6 present walking distance for the different conditions preoperatively as well as 1 and 2 years postoperatively. Tables 7, 8, 9, 10 and 11 show consumption of analgesics preoperatively and 1 and 2 years postoperatively, related to diagnosis for surgery. Patient-assessed satisfaction with surgical outcome after 1 and 2 years was none or less identical (Table 12). Tables 13, 14 and Fig. 33 present quality of life as measured by EQ-5D and by VAS. All patient groups experience a significant improvement in quality of life postoperatively.

**Table 1:** Pain on the VAS (mean), diagnosis-related

	Back			Leg		
	Preoperatively	1 year	2 year	Preoperatively	1 year	2 year
Disc herniation	46	22	25	66	19	22
Central stenosis	55	31	35	61	31	35
Lateral stenosis	51	31	31	62	34	32
Spondylolisthesis	59	27	29	52	26	25
DDD	62	29	32	42	22	25

**Table 2:** Walking distance, disc herniation (%)

	Preoperatively	1 year	2 year
<100 m	32	4	4
100 m–500 m	20	8	7
500 m–1 km	16	11	11
>1 km	32	77	78

**Table 3:** Walking distance, central spinal stenosis (%)

	Preoperatively	1 year	2 year
<100 m	41	18	21
100 m–500 m	30	20	20
500 m–1 km	14	17	15
>1 km	16	45	44

**Table 4:** Walking distance, lateral spinal stenosis (%)

	Preoperatively	1 year postop	2 years postop
<100 m	29	17	16
100 m–500 m	32	16	19
500 m–1 km	11	16	11
>1 km	28	51	54

**Table 5:** Walking distance, spondylolisthesis (%)

	Preoperatively	1 year postop	2 years postop
<100 m	17	5	9
100 m–500 m	28	13	12
500 m–1 km	13	13	15
>1 km	42	69	64

**Table 6:** Walking distance, DDD (%)

	Preoperatively	1 year postop	2 years postop
<100 m	11	4	5
100 m–500 m	19	9	7
500 m–1 km	24	16	15
>1 km	41	71	73

**Table 7:** Consumption of analgesics, disc herniation, preoperatively, 1 and 2 years postoperatively (%)

	Preoperatively	1 year postop	2 years postop
Regular	62	15	17
Intermittent	28	32	30
None	10	53	53

**Table 8:** Consumption of analgesics, central spinal stenosis preoperatively, 1 and 2 years postop (%)

	Preoperatively	1 year postop	2 years postop
Regular	53	28	31
Intermittent	31	33	32
None	16	40	37

**Table 9:** Consumption of analgesics, lateral spinal stenosis preoperatively, 1 and 2 years postop (%)

	Preoperatively	1 year postop	2 years postop
Regular	55	30	31
Intermittent	28	31	30
None	17	39	39

**Table 10:** Consumption of analgesics, spondylolisthesis preoperatively, 1 and 2 years postop (%)

	Preoperatively	1 year postop	2 years postop
Regular	44	23	25
Intermittent	28	30	28
None	28	48	47

**Table 11:** Consumption of analgesics DDD preoperatively, 1 and 2 years postop (%)

	Preoperatively	1 year postop	2 years postop
Regular	57	24	29
Intermittent	34	39	32
None	9	37	39

**Table 12:** Attitude toward surgical outcome 1 and 2 years postop, diagnosis-related

	1 year postop			2 years postop		
	Satisfied	Uncertain	Dissatisfied	Satisfied	Uncertain	Dissatisfied
Disc herniation	81	14	6	81	13	6
Central stenosis	66	24	10	64	22	13
Lateral stenosis	61	26	13	64	24	12
Spondylolisthesis	72	19	9	72	18	10
DDD	75	16	10	75	15	10

**Table 13:** EQ-5D means preoperatively, 1 year and 2 years postop, diagnosis-related

	Preop	1 year postop	2 years postop
Disc herniation	0.29	0.73	0.73
Central spinal stenosis	0.37	0.64	0.62
Lateral spinal stenosis	0.36	0.63	0.64
Spondylolisthesis	0.40	0.71	0.68
DDD	0.33	0.65	0.66

**Table 14:** EQ-VAS health assessment according to the VAS, means

	Preop	1 year postop	2 years postop
Disc herniation	46	73	73
Central spinal stenosis	48	65	63
Lateral spinal stenosis	50	65	66
Spondylolisthesis	52	72	72
DDD	42	67	66

### Oswestry disability index, ODI, preoperatively, 1 and 2 years post-operatively for all diagnoses

## Five-year follow-up of lumbar spine procedures

A total of 1,840 patients completed 1, 2 and 5-year follow-up after having undergone lumbar spine surgery in 2006. The most common diagnoses are disc herniation, 581 and central spinal stenosis, 706 patients. In all, 140 patients had been operated for lateral spinal stenosis, 130 for spondylolisthesis and 230 for segmental pain (DDD). The remaining 53 had other diagnoses (Table 15). Below is a comparison of several parameters at 1, 2 and 5-year follow-up. Only patients who responded on all four occasions are included.

**Table 15:** ODI results preoperatively, 1 and 2 years after lumbar spine surgery, diagnosis-related

	Preoperatively	1 year postop	2 years postop
Disc herniation	48	18	18
Central spinal stenosis	43	26	28
Lateral spinal stenosis	42	26	25
Spondylolisthesis	41	22	22
DDD	45	25	25

**Fig. 34:**
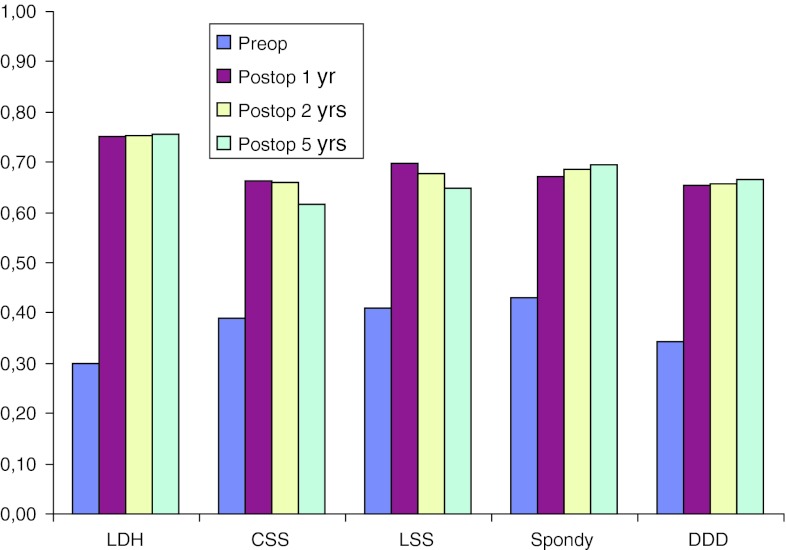
Quality of life preoperatively, 1, 2 and 5 years postoperatively, as measured by EQ-5D

Pain on the VAS, diagnosis-related, is remarkably stable over time (Table 16). Tables 17, 18, 19, 20 and 21 present walking distance after the different procedures preoperatively as well as 1, 2 and 5 years postoperatively. Tables 22, 23, 24, 25 and 26 show consumption of analgesics preoperatively and 1, 2 and 5 years postoperatively, related to diagnosis for surgery. Patient-assessed satisfaction with surgical outcome after 1, 2 and 5 years is more or less identical (Table 27). Tables 28, 29 and Fig. 34 present quality of life as measured by EQ-5D and by EQ-VAS. All patient groups experience a significant improvement in quality of life postoperatively.

**Table 16:** Pain on the VAS (mean), diagnosis-related

	Back				Leg			
	Preop	1 year	2 year	5 years	Preop	1 year	2 year	5 years
Disc herniation	42	21	22	22	63	19	20	20
Central stenosis	53	28	29	34	61	29	30	35
Lateral stenosis	53	28	28	31	62	31	29	33
Spondylolisthesis	56	25	26	28	52	24	24	24
DDD	62	31	29	30	45	22	22	22

**Table 17:** Walking distance, disc herniation (%)

	Preoperatively	1 year	2 year	5 years
<100 m	32	4	5	5
100 m–500 m	22	7	7	5
500 m–1 km	17	8	9	9
>1 km	29	81	79	81

**Table 18:** Walking distance, central spinal stenosis (%)

	Preoperatively	1 year	2 year	5 years
<100 m	40	16	17	22
100 m–500 m	33	17	17	17
500 m–1 km	13	16	15	16
>1 km	15	51	52	44

**Table 19:** Walking distance, lateral spinal stenosis (%)

	Preoperatively	1 year	2 year	5 years
<100 m	22	7	10	16
100 m–500 m	33	11	10	10
500 m–1 km	16	20	18	18
>1 km	29	62	62	57

**Table 20:** Walking distance, spondylolisthesis (%)

	Preoperatively	1 year	2 year	5 years
<100 m	16	4	5	6
100 m–500 m	24	19	11	11
500 m–1 km	20	12	12	12
>1 km	40	76	71	72

**Table 21:** Walking distance, DDD (%)

	Preoperatively	1 year	2 year	5 years
<100 m	9	5	6	5
100 m–500 m	23	10	8	9
500 m–1 km	26	14	12	9
>1 km	42	72	74	77

**Table 22:** Consumption of analgesics, disc herniation, preoperatively, 1, 2 and 5 years postoperatively (%)

	Preoperatively	1 year	2 year	5 years
Regular	59	16	17	15
Intermittent	29	28	29	33
None	13	56	54	52

**Table 23:** Consumption of analgesics, central spinal stenosis preoperatively, 1, 2 and 5 years postop (%)

	Preoperatively	1 year	2 year	5 years
Regular	48	23	26	29
Intermittent	33	33	34	32
None	19	45	40	39

**Table 24:** Consumption of analgesics, lateral spinal stenosis preoperatively, 1, 2 and 5 years postop (%)

	Preoperatively	1 year	2 year	5 years
Regular	49	23	27	27
Intermittent	26	33	32	29
None	25	44	41	44

**Table 25:** Consumption of analgesics, spondylolisthesis preoperatively, 1, 2 and 5 years postop (%)

	Preoperatively	1 year	2 year	5 years
Regular	40	20	23	24
Intermittent	39	33	33	29
None	21	47	44	48

**Table 26:** Consumption of analgesics DDD preoperative, 1, 2 and 5 years postop (%)

	Preoperatively	1 year	2 year	5 years
Regular	51	25	24	26
Intermittent	36	36	38	35
None	14	40	38	39

**Table 27:** Attitude toward surgical outcome 1, 2 and 5 years postop, diagnosis-related

	1 year postop			2 years postop			5 years postop		
	Satisfied	Uncertain	Dissatisfied	Satisfied	Uncertain	Dissatisfied	Satisfied	Uncertain	Dissatisfied
Disc herniation	80	16	5	81	14	5	83	11	6
Central stenosis	70	21	10	68	20	12	66	21	13
Lateral stenosis	73	18	7	70	20	11	69	21	10
Spondylolisthesis	80	16	5	82	12	6	83	6	11
DDD	76	17	7	75	17	8	77	14	9

**Table 28:** EQ-5D means preoperatively, 1, 2 and 5 years postop, diagnosis-related

	Preoperatively	1 year postop	2 years postop	5 years postop
Disc herniation	30	75	75	76
Central stenosis	39	66	66	62
Lateral stenosis	41	70	68	65
Spondylolisthesis	43	67	69	69
DDD	34	65	66	66

**Table 29:** EQ-5D health assessment according to the VAS, means

	Preoperatively	1 year postop	2 years postop	5 years postop
Disc herniation	47	74	74	74
Central stenosis	52	67	65	62
Lateral stenosis	52	70	70	66
Spondylolisthesis	52	70	70	71
DDD	48	65	67	66

## Surgery for degenerative cervical spine disease

In 2011, 698 patients were included in the register after surgery for degenerative cervical spine disease, including 53 % men and 47 % women. In all, 20 % of the patients were smokers and 10 % had previously undergone cervical spine surgery.

Preoperative duration of pain was as follows: <3 months 2 %, 3–12 months 24 %, 1–2 years 20 % and more than 2 years 45 %, while 9 % denied any neck pain. Patients experienced radiation of pain to the arm(s) as follows: 4 % of patients for <3 months, 32 % for 3–12 months, 24 % for 1–2 years and 33 % for more than 2 years, while 7 % denied any arm pain.

Regular consumption of analgesics was confirmed by 53 % of patients, intermittent by 30 % and none by the remaining 17 %.

Estimated walking distance was reported by 13 % of patients to be <100 m, 12 % 100–500 m, 16 % 500 m–1 km and 59 % >1 km. In all, 75 % reported subjective deterioration of fine motor function in their hands.

Co-morbidity was reported in the form of heart disease 2 %, neurological disease 3 %, cancer 0 %, other disease affecting ability to walk 9 %, or other disease causing pain 13 %, while 72 % denied any co-morbidity.

Mean neck pain on the VAS was 55 with a spread from 0 to 100. The corresponding figures for arm pain were 53 with a spread from 0 to 100.

Mean preoperative EQ-5D index value was 0.38 for patients, while the results of the Neck Disability Index (NDI) were as follows: mean 62.6. Distribution on the European myelopathy score was 15.11.

### Surgical data

In all, 44 % of the patients were operated for cervical disc herniation, 26 % for cervical spinal stenosis, 23 % for cervical foraminal stenosis, 1.48 % for segmental neck pain, 1.9 % for rheumatoid arthritis and 0.1 % for ankylosing spondylitis; 3.2 % were operated for some other diagnosis.

With respect to the clinical presentation, 12 % of patients had no neurological findings, 59 % radicular involvement, 23 % medullary involvement and the remaining 6 % combined radicular and medullary involvement.

Horizontal instability between C1 and C2 was seen in 2 % of cases, vertical between C0 and C2 in <1 % of cases and subaxial instability between C2 and Th1 in 2.7 % of cases. Combined instability was assessed to be present in 0.6 % of cases.

Surgical interventions were performed as follows:

- Disk removal without fusion <1 %
- Disc removal with fusion without plate 2 %
- Disc removal with fusion with plate 9 %
- Disc removal with fusion cage without plate 20 %
- Disc removal with fusion cage with plate 35 %
- Corpectomy 7 %
- Disc replacement 5 %
- Laminectomy without fixation 4 %
- Laminectomy with fixation 6 %
- Laminoplasty <1 %
- Foraminotomy 6 %
- Combination laminectomy/foraminotomy 2 %
- Posterior fixation without decompression 2 %
- Other procedure without implant <1 %, and
- Other procedure with implant 2 %.

Anterior implant was used in 80 % of cases and posterior in 10 % of cases.

### Follow-up data

About 76 % of the 620 patients operated in 2010 also had 1-year follow-up. Average preoperative NDI was 63 and postoperative 47. Radiculopathy/arm pain improved from an average of 48 on the VAS preoperatively to an average of 26 postoperatively.

Corresponding subjective scoring of change in arm pain 1 year postoperatively: greatly improved 53 %, somewhat improved 18 %, unchanged 23 and 7 % perceived worsening.

Patient assessment of change in walking distance 1 year postoperatively: >100 m 9 %, 100–500 m 12 %, 0.5–1 km 14 % and >1 km 64 %.

Quality of life as measured by EQ-5D improved from mean 0.39 preoperatively to 0.64 postoperatively at 1 year.

## Spine fracture surgery

This diagnostic category has been recently added to Swespine and totally 2,299 fractures have been recorded. However, only limited and mainly preoperative data are available to date.

In 2011, 423 operations were registered for spinal column fractures. The majority of patients subjected to surgery for vertebral fractures belonged to the age group 60–69 years, and 65 % were male. In all, 22 % of patients operated had some degree of neurological damage, and 92 % of the procedures registered were carried out at university hospitals. According to AO classification, 31 % of the fractures were type A, 46 % type B and 23 % type C (Table 30).

**Table 30:** Fracture types according to AO classification (%)

Class A	Class B	Class C
31	46	23

The single largest group of fractures in the register involved Th11–L2 fractures. Of the fractures registered, 86 % were operated with posterior fusion with or without decompression and 4 % with vertebroplasty. Even here, the most common age group was 60–69 years, but these fractures also have a clear peak at age 20–29 years as they include both high-energy injuries in younger and middle-aged patients and osteoporotic fractures in older patients.

Neurological involvement in the form radiculopathy was seen in 20 % of cases and in the form myelopathy in 21 % of cases with the following distribution according to the Frankel Scale: A 28 %, B 9 %, C 19 %, D 24 % and E 20 % (Table 31).

**Table 31:** Neurological function according to the Frankel Classification system (%)

Classification	Percent
A	28
B	9
C	19
D	24
E	20

Two years after surgery, 72 % of the patients were satisfied with the outcome of the procedure, 21 % uncertain and 6 % dissatisfied. However, many of the patients probably had no or very moderate back pain before the fracture and have difficulty assessing what the status would have been without surgery. Of those who worked before the fracture, 38 % returned to work full-time and 15 % had returned to work part-time. In all, 29 % of patients took analgesics regularly and 33 % occasionally. The mean EQ-5D index value was 0.66 2 years after the procedure.

## Surgery for spinal metastases

This diagnostic category has also been recently added to Swespine and contains totally 794 operations for spinal metastasis. To date, only limited and mainly preoperative data are available.

In all, 211 patients were registered for spinal metastasis surgery in 2011. 8 % of the patients were smokers. Indications for surgery are as follows: neurological involvement 53 %, back/leg pain 14.5 %, progressive deformity 1.4 %, neurological involvement + back/leg pain 18.8 %, neurological involvement + progressive deformity 2.2 %, back + progressive deformity 3.6 %, neurological involvement + back + progressive deformity 6.5 %. For the remaining 34.6 %, the indication for surgery was not reported.

The primary tumor was known in 72 % of cases and unknown in 28 %. Among known primary tumors, the following were most common: prostate 41 %, breast 9.8 %, kidney 3.9 %, thyroid 1 %, lung 10.8 %, blood-forming organs 12.7 %, GI tract 2.9 % and other 17.6 % (Table 32).

**Table 32:** Primary tumor at spinal metastasis (%)

Primary tumor	Percent
Prostate	41
Lung	11
Breast	10
Kidney	4
GI tract	3
Blood-forming organs	13
Thyroid	1
Other known primary tumor	18
Unknown primary tumor	28

In 41.8 % of cases, a pathologic fracture was diagnosed. Neurological involvement was distributed as follows on the Frankel Scale: A 6 %, B 6.7 %, C 32.8 %, D 31.3 % and E 23.1 %. Preoperative analgesic consumption was as follows: 81.9 % morphine analgesics, 13.4 % non-morphine analgesics and 4.7 % no analgesic consumption.

Surgical procedures included posterior and anterior decompression as well as possible fusion. In all, 90 % had posterior decompression at the following levels: cervical, thoracic and lumbar levels, while 10 % had anterior decompression at the following levels: cervical, thoracic and lumbar. Fusion was carried out in 39 % of cases.

Resection of the tumor was carried out in 84 % of cases; in 5 % of cases as wide excision, 19 % marginal excision, and 76 % intralesional excision.

## Analysis of disc replacement surgery of the lumbar spine

### Introduction

While quite common in European continental countries and subsequently in the US, only a few total disc replacements (TDR) were carried out in Sweden in the 1990s, but they are not included in the register. TDR has been performed more routinely and systematically in Sweden since 2003. Little scientific documentation is available. Two randomized FDA studies in the US have been published. However, their results have been strongly disputed and it is doubtful whether these results can be applied to Swedish conditions.

TDR in Sweden has been evaluated in a randomized study published in a thesis in 2010 with 2-year follow-up (clinical results by Berg et al. 2010, and in a cost-effectiveness analysis by Fritzell et al. 2011).

### Material

**Fig. 35:**
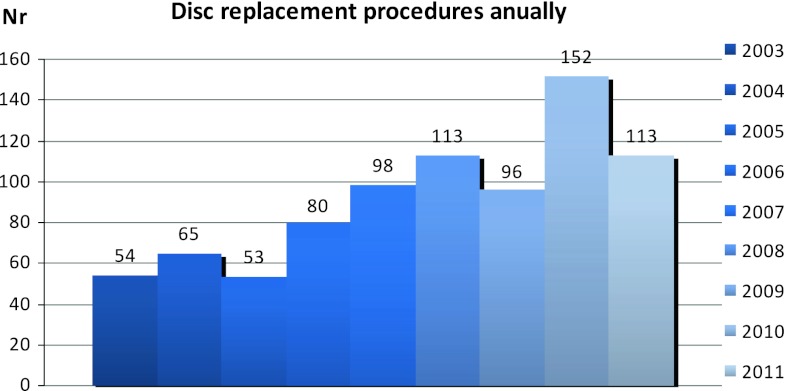
Number of disc replacement procedures annually, 2003–2011

A total of 879 disc replacements in the lumbar spine have been registered in our database through the end of September 2012. Figure 35 shows the number of procedures performed annually.

The diagnoses entered in the register are as follows: segmental pain 834, paramedian disc herniation 17, central disc herniation 11, postoperative instability 8, central spinal stenosis 3, isthmic spondylolisthesis 2, other diagnosis 2, and no information about diagnosis in two cases.

The majority of operations (773) were carried out at one center and the remainder at five different centers.

This analysis compares the 879 disc replacements with 3,066 fusions carried out during the same time period. Follow-up data for at least 1 year were available for 670 disc replacements and 2,517 fusions. Table 33 presents follow-up rate at 1 year (FU1), 2 years (FU2) and 5 years (FU5).

**Table 33:** Follow-up rate FU 1 year, FU 2 years and FU 5 years (%)

Time	Fusion (*n* = 2,517)			Disc replacement (*n* = 670)		
	Followed up	Missing	FU %	Followed up	Missing	FU %
FU1	1,914	603	76	561	109	84
FU2	1,399	745	65	388	133	74
FU3	603	502	0.56	165	0.56	75

The Full-time sick leave after surgery is consistently higher for TDR, probably due to the previously mentioned dissertation project carried out during the period. Table 34 shows baseline data. Significant differences between disc replacement and fusion patients can be seen in several regards.

**Table 34:** Baseline-data

	Fusion	Disc replacement	
	%	%	*χ* ^2^-test
Woman	53	50	ns
Smokers	16	12	<0.01
Previous back surgery	37	21	<0.001
Full-time sick leave	43	37	0.002
Duration of symptoms <6 months	23	30	0.002
Duration of symptoms <12 mos	78	79	ns
Other disease	21	15	ns
Pt believes in return to employment	53	75	<0.001
	Unit	Unit	Mann–Whitney/*T* test
VAS back pain	64	61	<0.01
EQ5D	0.3	0.4	<0.001
ODI	46	41	<0.001
Age	46	40	<0.001
BMI	26	25	<0.01

### Results

The results are presented in five different ways:

1. Global assessment, which means that the patient answers the question “How is your back pain today compared with before surgery?” and we have calculated the proportion of patients who state they are “pain-free or significantly improved”.
2. Full-time sick leave after surgery.
3. Patient satisfaction with the surgical outcome by asking the question “What is your opinion of the surgical outcome?” with response options “Satisfied, uncertain, dissatisfied”.
4. Change in quality of life as measured by EQ-5D.
5. Changes in back pain as measured by VAS.

Tables 35, 36, 37, 38 and 39 present the results. A significant difference, in favor of disc replacement surgery, was found in all measurements using the global assessment and the VAS for back pain. No significant difference was found at 5-year follow-up regarding satisfaction with results, nor was any significant difference found in any of the measurements concerning changes in quality of life.

**Table 35:** Improvement of back pain as measured by global assessment (%)

Time	Fusion	TDR	*χ* ^2^-test
FU1	58	68	<0.001
FU2	59	71	<0.001
FU3	58	69	<0.001

**Table 36:** Full-time sick leave after surgery (%)

Time	Fusion	TDR	*χ* ^2^-test
FU1	20	7	<0.001
FU2	15	7	<0.001
FU3	8	8	ns

**Table 37:** satisfied with the surgical outcome (%)

Time	Fusion	TDR	*χ* ^2^-test
FU1	69	77	<0.001
FU2	71	7S	<0.001
FU3	69	75	ns

**Table 38:** Change in quality of life as measured by EQ-5D

Time	Fusion	TDR	Mann–Whitney *T*-test
FU1	0.28	0.31	ns
FU2	0.29	0.3	ns
FU3	0.28	0.31	ns

**Table 39:** Change in back pain as measured by visual analog (VAS)

Time	Fusion	TDR	Mann–Whitney *T*-test
FU1	−29	−35	<0.001
FU2	−29	−33	<0.01
FU3	−28	−34	<0.04

**Fig. 36:**
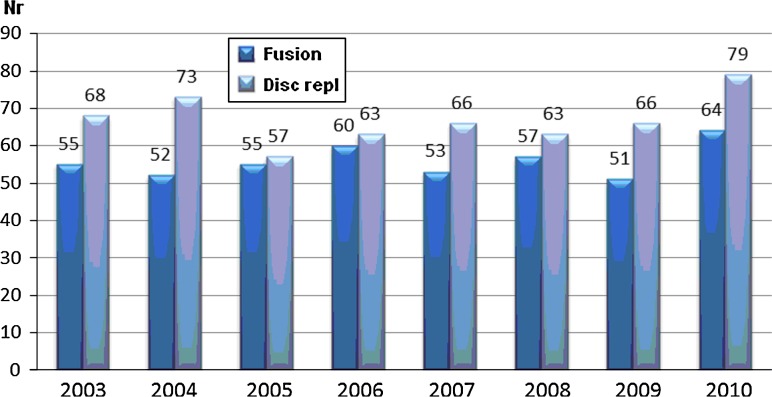
Improvement of back pain as measured by Global Assessment

Figure 36 measures the rate of the responses “Pain-free/Significantly improved” on an annual basis at 1-year follow-up to ascertain whether any change in outcome occurred over time. No clear trends regarding changes were found when comparing fusion and disc replacement surgery.

Table 40 compares the two surgical methods regarding the proportion of patients who state that they are worse at 1-year and 2-year follow-up than they were prior to surgery. The comparison shows a trend toward fewer patients who rate their status as worse after disc replacement surgery than after fusion.

**Table 40:** Worsening of back pain measured by global assessment(=worse) by year at FU 1 year

Surgery year	Fusion		Disc replacement	
	FU l year	FU 2 years	FU l year	FU 2 years
2003	6	5	2	0
2004	8	5	2	4
2005	8	8	9	6
2006	7	7	0	1
2007	8	6	3	2
2008	8	6	5	5
2009	5	5	4	3
2010	6	3	3	0

### New index surgery and re-intervention

The term “new index surgery” refers to a new operation carried out to address a new diagnosis in a different segment from prior surgery. Reoperation refers to a repeat procedure in the previously operated segment. In the fusion group, 457 of 3,066 (15 %) patients underwent a new fusion procedure in an adjacent segment. A new disc replacement procedure was carried out in 79 of 879 cases (9 %).

Tables 41 and 42 present data about re-intervention after disc replacement surgery. The type of operation carried out in the group “Other procedure” cannot be ascertained from the register, but in the majority of cases likely refers to posterior surgical fusion. A total of 28 re-interventions (3 %) were carried out. In the fusion group, 427 reoperations (14 %) were carried out, including 226 surgeries with removal of implant. If these are excluded, the remaining 201 (7 %) reoperations were carried out because of complications.

**Table 41:** Reoperation after primary TDR

Reason	Number
Repositioning of prosthesis	4
Removal of prosthesis	1
Reoperation of dural damage	1
Other procedure	22

**Table 42:** Reoperation because of complication

	Number of reop	%
Fusion, reop total	427	14
Fusion, implant removal	226	7.4
Fusion, other reop	201	6.6
Disc replacement	28	3

In Tables 43 and 44, baseline data suspected of influencing surgical outcome were assessed at all three follow-ups using a multivariate regression analysis, both in relation to global assessment and in relation to satisfaction with surgical outcome. Surgical procedure (disc replacement or fusion) was entered as an independent variable. Several of the variables correlated significantly at several follow-ups, but surgical procedure showed no significant correlation at any of the follow-ups. Previous back surgery, ODI and the patient’s own belief in the possibility of returning to work postoperatively correlated significantly with the results at all three follow-ups.

**Table 43:** Multivariate regression analysis of factors with possible influence on surgical outcome

	FU1		FU2		FU5	
	OR	*P*	OR	*P*	OR	*P*
Men	0.74	0.001	–	ns	–	ns
Smokers	–	ns	–	ns	2	0.002
Previous back surgery	1.8	<0.001	1.6	<0.001	1.6	0.006
Duration of symptoms	1.3	<0.001	1.4	<0.001	1.6	0.02
Age	–	ns	–	ns	–	ns
Does not expert to return to work	1.2	<0.001	1.3	<0.001	1.2	0.002
Surgical technique	–	ns	–	ns	–	ns
ODI	1.02	<0.001	1.02	<0.001	1.03	<0.001

Dependent variable = Global Assessment (0 = pain-free/significantly improved, 1 = not pain-free/significantly improved)

Follow-up Fusion: FU1 year: 1,725, FU2 years: 1,285, FU5 years: 545

Follow-up Disc Replacement: FU1 year: 575, FU2 years: 424, FU5 years: 197

**Table 44:** Multivariate regression analysis of factors with possible influence on surgical outcome

	FU1		FU2		FU5	
	OR	*P*	OR	*P*	OR	*P*
Men	0.7	<0.001	0.7	0.004	–	ns
Smokers	–	ns	1.4	0.03	–	ns
Previous back surgery	1.8	<0.001	1.4	0.005	1.8	0.001
Duration of symptoms	1.3	0.006	1.4	0.002	–	ns
Age	–	ns	–	ns	–	ns
Does not expert to return to work	1.1	<0.001	1.2	<0.001	–	ns
Surgical technique	–	ns	–	ns	–	ns
ODI	1.02	<0.001	1.02	<0.001	1.03	<0.001

Dependent variable = (“Satisfied with surgical outcome” (0 = Yes, 1 = No)

Follow-up Fusion: FU1 year: 1,698, FU2 years: 1,276, FU5 years: 540

Follow-up Disc Replacement: FU1 year: 572, FU2 years: 421, FU5 years: 195

### Discussion

The documentation and follow-up rate are good for the results reported at 1- and 2-year follow-up, while the statistical base is smaller for the 5-year follow-up, which is why the interpretation of 5-year results is much more uncertain. However, the results at 1 and 2 years for patients who undergo disc replacement are significantly better in many respects than for patients who undergo fusion surgery. The finding that there was no difference in change (improvement) of quality of life may be explained by the fact that disc replacement patients begin at a higher level and therefore end at a higher level of quality of life. Also in regard to capacity to work, disc replacement patients fare better than fusion patients.

The multivariate analysis also shows that the surgical procedure seems to be less important than several individual-dependent factors. Nevertheless, the surgical method should not be construed as irrelevant. However, it does express the differences in case mix between the two surgical groups. Patients who are candidates for disc replacement are a subgroup among those diagnosed with segmental pain with other prognostic factors than patients who are candidates for fusion. There is a selection process before surgery which most likely influences the outcome in favor of TDR.

The results support the conclusion that TDR works as well as fusion in patients with lumbar pain due to degenerative disc disease. However, it must be underscored that patient selection appears to be more important than surgical method, and that TDR candidates have a better initial status than fusion patients as a group. This assessment also applies only to 1-year follow-up. Data from subsequent follow-ups are still insufficient. Problems with reoperations in the aftermath of surgical procedures for DDD, regardless of method, can still be seen and have not yet been resolved. It should also be noted that most TDR surgeries were performed at one clinic by the same surgeon, which is why the generalizability of these results must be questioned.

TDR may be a viable alternative to fusion in a small group of patients with chronic low back pain who meet strict selection criteria; however, the time perspective is definitely a matter of concern, and the final comparison also cannot be based solely on registry data, but also requires prospective randomized studies.

## Number of registered operations and follow-up rate

**Fig. 37:**
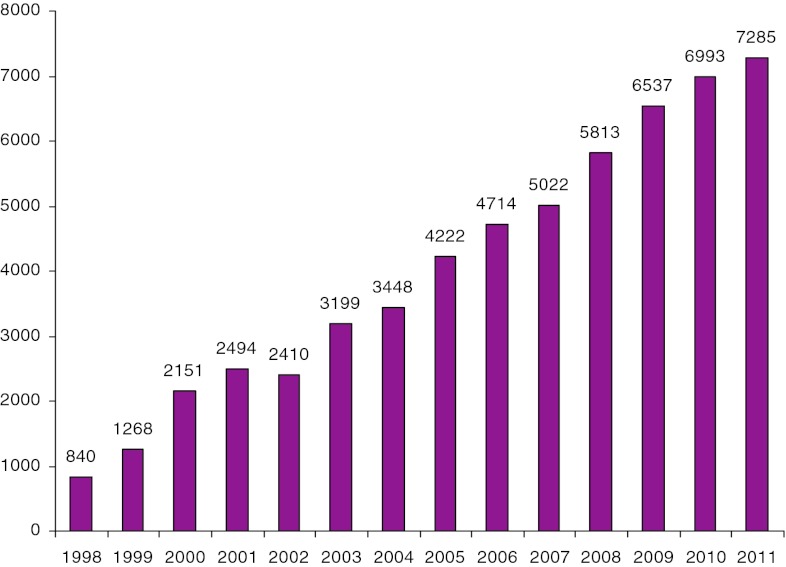
Number of patients entered in the register for degenerative disorders of the lumbar spine 1999–2011

The number of patients entered in the surgery register for degenerative lumbar disorders has steadily increased in recent years, as illustrated in Fig. 37.

The increase is mainly due to more complete registration within Swespine over time but, also, due to a slight increase in surgical lumbar spine interventions. The annual number of operations for degenerative lumbar disorders (mainly spinal stenosis and disc herniation) is approximately 8,000 which means that reporting today covers 90 % of the operations.

**Fig. 38:**
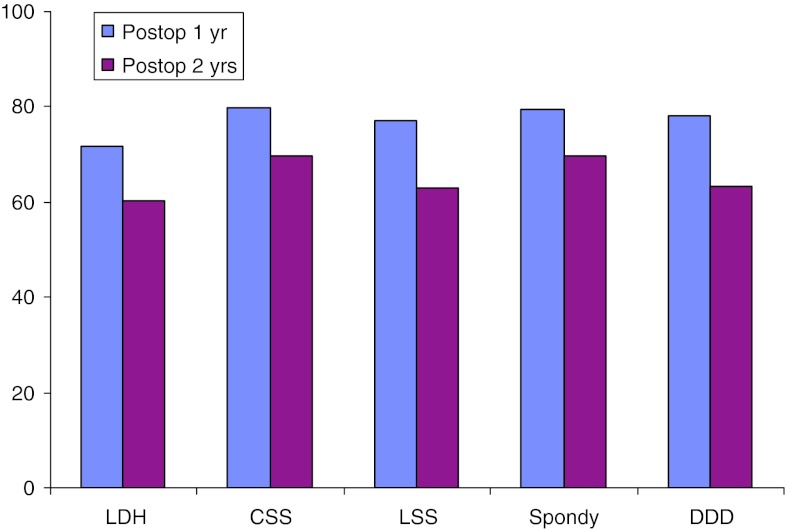
Current follow-up rate

The follow-up rate has been consistent over the last years and amounts to 79 % at 1 year and 67 % at 2 years. Figure 38 shows the follow-up rate at 1 and 2 years for patients operated in 2009.

## Concluding remarks

The last decade has witnessed an enormous increase in research concerning spinal disorders and the outcome of spinal surgery. Also, a very high number of new implants and new techniques have been introduced on the market, some of which have gained a place in the surgical armamentarium and some of which have disappeared again.

For the introduction of new methods and techniques, basic studies such as biomechanical testing, biochemical investigations, etc. are required. They should be followed by pilot studies and, after that, randomized-controlled trials comparing the new technique to the existing golden standard for the treatment modality in question.

The final proof of the value of the new technique is documenting its effect when implemented in general practice, i.e. when it is utilized by spine surgeons in general. Here, broad registrations like local and national registers are important for giving us knowledge in this aspect. Other benefits from large registries are the possibilities to achieve quality assurance and observing trends and changes over time. Also, the documentation of the effect of a surgical procedure in the long-term is possible to evaluate. Due to this fact, an increasing interest has focused on large registries; and Swespine is among those being on the scene for the longest time and also being most disseminated.

Another issue that registers can provide is international comparisons. Swespine has been recently adopted in Denmark (Danespine), Iceland (Icespine) and the Netherlands. Several other countries are interested in negotiating a collaboration of the same type. Other registers can be exemplified by Spine Tango, administered by the Spine Society of Europe, which already has several centers in Europe delivering data. Other registers, as the Norwegian spine register and the Singapore register, are examples of comprehensive and successful registers.

For this purpose, an international meeting on Spine registries is planned in conjunction with the upcoming meeting of the International Society for the Study of the Lumbar Spine (ISSLS) in Scottsdale, Arizona May 2013. If for example a common platform of baseline data (core data set) could be agreed upon, international comparisons would be strongly facilitated. The authors of this report, the Swespine Steering Group welcome all interest and contributions to this work, and we welcome all interested parties to Scottsdale on May 12; http://www.issls.org/home.aspx.

## References

[1] Strömqvist B, Jönsson B (1993). Computerized follow-up after surgery for degenerative lumbar spine diseases. Acta Orthop Scand.

[2] Jönsson B, Strömqvist B (1998). Ländryggskirurgi: Registret kan räddas. Ortopediskt Magasin.

[3] Jönsson B, Strömqvist B (1999). Significance of a persistent positive straight leg raising test after lumbar disc surgery. J Neurosurg.

[4] Strömqvist B, Jönsson B, Zanoli G (1999). The significance of VAS in evaluating pain outcomes of spine surgery. A prospective, consecutive study of 755 operated patients. Eur Spine J.

[5] Strömqvist B, Jönsson B (2000). Det nationella registret blir alltmer fullständigt. Dagens Medicin Nr.

[6] Svensk Ryggkirurgisk Förenings registergrupp (2000) Uppföljning av ländryggskirurgi i Sverige 1999. Rapport. pp 21

[7] Zanoli G, Strömqvist B (2000). Lessons learned searching for a HRQoL instrument to assess the results of treatment in persons with lumbar disorders. Spine.

[8] Padua R, Strömqvist B, Jönsson B, Romanini E, Zanoli G (2000). Imparare dagli errori del passato in chirurgia vertebrale: registro nazionale svedese e studi multicentrici italiani. Ital J Orthop Trauma.

[9] Strömqvist B, Jönsson B, Fritzell P, Hägg O, Larsson B-E, Lind B (2001). The Swedish national register for lumbar spine surgery. Acta Orthop Scand.

[10] Zanoli G, Strömqvist B, Jönsson B (2001). Visual analog scales for interpretation of back and leg pain intensity in patients operated for degenerative lumbar spine disorders. Spine.

[11] Svensk Ryggkirurgisk Förenings registergrupp (2001) Uppföljning av ländryggskirurgi i Sverige 2000 Report. pp 21

[12] Svensk Ryggkirurgisk Förenings registergrupp (2002) The national Swedish register for lumbar spine surgery. Report 2001. pp 30

[13] Strömqvist B (2002). Evidence-based lumbar spine surgery. The role of national registration. Acta Orthop Scand.

[14] Zanoli G, Strömqvist B, Jönsson B, Padua R, Romanini E (2002). Pain in low-back pain. Problems measuring outcomes in musculoskeletal disorders. Acta Orthop Scand.

[15] Svensk Ryggkirurgisk Förenings registergrupp (2003) Uppföljning av ländryggskirurgi i Sverige 2002. Report. pp 26

[16] Svensk Ryggkirurgisk Förenings registergrupp (2004) Uppföljning av ländryggskirurgi i Sverige 2003. Report. pp 24

[17] Jansson KÅ (2005) On lumbar spinal stenosis and disc herniation surgery. Thesis, Department Surgical Sciences, Section Orthopedics, Karolinska Institute, Stockholm

[18] Jansson KÅ, Németh G, Granath F (2005). Health-related quality of life in patients before and after surgery for a herniated lumbar disc. J Bone Joint Surg.

[19] Zanoli G (2005) Outcome assessment in lumbar spine surgery. Thesis, Department of Orthopedics, Lund University

[20] Fritzell P (2005). Fusion as treatment for chronic low back pain—existing evidence, the scientific frontier and research strategies. Eur Spine J.

[21] Svensk Ryggkirurgisk Förenings registergrupp (2005) Uppföljning av ländryggskirurgi i Sverige 2004. Rapport. pp 24

[22] Fritzell P, Strömqvist B, Hägg O (2006). A practical approach to spine registers in Europe. The Swedish experience. Eur Spine J.

[23] Strömqvist B, Fritzell P, Hägg O, Jönsson B, Swedish Society of Spinal Surgeons (2005). One-year report from the Swedish National Spine Register. Swedish Society of Spinal Surgeons. Acta Orthop.

[24] Strömqvist B, Fritzell P, Hägg O, Jönsson B (2006) Lägesrapport om svenska nationella ryggregistret. Ortopediskt Magasin (2): 9–10,12

[25] Svensk Ryggkirurgisk Förenings registergrupp (2006) Uppföljning av ländryggskirurgi i Sverige 2005. Report

[26] Zanoli G, Nilsson LT, Strömqvist B (2006). Reliability of the prospective data collection protocol of the Swedish Spine Register. Test-retest analysis of 119 patients. Acta Orthop.

[27] Zanoli G, Strömqvist B, Jönsson B (2006). SF-36 scores in degenerative lumbar spine disorders: analysis of prospective data from 451 patients. Acta Orthop.

[28] Strömqvist B, Hedlund R, Jönsson B, Tullberg T (2007). Ländryggens sjukdomar. Läkartidn.

[29] Strömqvist F, Ahmad M, Strömqvist F, Hildingsson C, Jönsson B (2008). Lumbar disc herniation surgery and gender-related differences. Touch Briefings.

[30] Strömqvist F, Ahmad M, Hildingsson C, Jönsson B, Strömqvist B (2008). Gender differences in lumbar disc herniation surgery. Acta Orthop.

[31] Strömqvist B, Fritzell P, Hägg O, Jönsson B, Swedish Society of Spinal Surgeons (2009). The Swedish spine register: development, design and utility. Eur Spine J.

[32] Berg S, Tullberg T, Branth B, Olerud C, Tropp H (2009). Total disc replacement compared to lumbar fusion: a randomised controlled trial with 2-year follow-up. Eur Spine J.

[33] Strömqvist B, Fritzell P, Hägg O, Jönsson B Svensk Ryggkirurgisk Förening (2010) Uppföljning av ländryggskirurgi i Sverige. Report år 2009. pp 51. ISBN 978-91-978553-0-3

[34] Strömqvist B, Fritzell P, Hägg O, Jönsson B. Swedish Society of Spinal Surgeons (2010) The Swedish Spine Register. The 2009 report. pp 58. ISBN 978-91-978553-1-0 10.1080/17453690510041950 16234179

[35] Berg S (2010) On total disc replacement. Thesis. Linköping University

[36] Strömqvist F, Jönsson B, Strömqvist B (2010). Dural lesions in lumbar disc herniation surgery: incidence, risk factors, and outcome. Eur Spine J.

[37] Sandén B, Försth P, Michaëlsson K (2011). Smokers show less improvement than nonsmokers two years after surgery for lumbar spinal stenosis: a study of 4555 patients from the Swedish spine register. Spine.

[38] Fritzell P, Brisby H, Hägg O (2011). The national qualite regristries: long and complicated way if the medical profession doesn’t see the advantages. Läkartidn.

[39] Fritzell P, Berg S, Borgstrom F, Tullberg T, Tropp H (2011). Cost effectiveness of disc prosthesis versus lumbar fusion in patients with chronic low back pain: randomized controlled trial with 2-year follow-up. Eur Spine J.

[40] Ohrn A, Olai A, Rutberg H, Nilsen P, Tropp H (2011). Adverse events in spine surgery in Sweden: a comparison of patient claims data and national quality register (Swespine) data. Acta Orthop.

[41] Sigmundsson FG, Kang XP, Jönsson B, Strömqvist B (2011). Correlation between disability and MRI findings in lumbar spinal stenosis. A prospective study of 109 patients operated on by decompression. Acta Orthop.

[42] Strömqvist B, Fritzell P, Hägg O, Jönsson B, Sandén B (2012). Swespine—en lägesrapport. Långvarig smärta och rökning ger dåligt resultat. Ortopediskt Magasin.

[43] Strömqvist F, Jönsson B, Strömqvist B (2012). Dural lesions in decompression for lumbar spinal stenosis—incidence, risk factors and effect on outcome. Eur Spine J.

[44] Fritzell P, Ohlin O, Borgström F (2011). Cost-effectiveness of Balloon Kyphoplasty (BKP) vs. Standard medical treatment in patients with osteoporotic vertebral compression fracture—a Swedish multicenter RCT with 2-year follow up. Spine.

[45] Strömqvist B, Fritzell P, Hägg O, Jönsson B, Sandén B (2012). Swespine—en lägesrapport. Långvarig smärta och rökning ger dåligt resultat. Ortopediskt Magasin.

[46] Strömqvist B, Fritzell P, Hägg O, Jönsson B. Swedish Society of Spinal Surgeons (2012) Swespine. The Swedish Spine Register. The 2011 Report. ISBN 978-91-979378-8-7

[47] Sigmundsson FG, Kang XP, Jönsson B, Strömqvist B (2012). Prognostic factors in lumbar spinal stenosis surgery—a prospective study of imaging and patient related factors in 109 patients operated on by decompression. Acta Orthop.

[48] Knutsson B, Michaëlsson K, Sandén B (2013). Obesity is associated with inferior results after surgery for lumbar spinal stenosis: a study of 2633 patients from the Swedish Spine Register. Spine.

[49] Robinson Y, Michaëlsson K, Sandén B (2013). Instrumentation in lumbar fusion improves back pain but not quality of life 2 years after surgery. Acta Orthop.

[50] Fritzell P, Hägg O, Jönsson B, Strömqvist B. Surgery for lumbar disc herniation—factors of importance for outcome after 1 and 2 years. Analysis of data from Swespine—the Swedish national spine register, Spine (in press)

[51] Strömqvist B, Berg S, Gerdhem P, Johnsson R, Möller A, Sahlstrand T, Ahmed S, Tullberg T. X-Stop versus decompressive surgery for lumbar neurogenic intermittent claudication—a randomized controlled trial with 2 years follow-up, Spine (in press) 10.1097/BRS.0b013e31828ba413 23403549

